# Aip mutation leads to significant alterations in gene expression, altered sensitivity to Ahr ligands, and early lethality in zebrafish 

**DOI:** 10.3389/ftox.2026.1875384

**Published:** 2026-07-06

**Authors:** Dante M. Perone, Jane K. La Du, Sibel I. Karchner, Neelakanteswar Aluru, Spencer R. Stinson, Lisa Truong, Mark E. Hahn, Robyn L. Tanguay

**Affiliations:** 1 Sinnhuber Aquatic Research Laboratory and the Department of Environmental and Molecular Toxicology, Oregon State University, Corvallis, OR, United States; 2 Department of Biology, Woods Hole Oceanographic Institution, Woods Hole, MA, United States

**Keywords:** PCB126, 5-nitroacenaphthene, AhR (aryl hydrocarbon receptor), BkF, differential susceptibility, gene set enrichment analyses (GSEA), leflunomide, RNA seq analysis

## Abstract

Humans and other organisms are regularly exposed to chemical compounds that elicit toxicity through the aryl hydrocarbon receptor (AHR) signaling pathway. AHR agonists include polycyclic aromatic hydrocarbons, polychlorinated biphenyls, and some natural products, pharmaceuticals, and endogenous compounds. Interindividual differences in sensitivity to AHR agonists exists. Several populations of Atlantic killifish (*Fundulus heteroclitus*) have independently evolved resistance to high levels of pollution. Genome-wide association studies suggested that variation in the AHR interacting protein (AIP) was associated with resistance to AHR ligands. AIP binds to AHR in the cytosol where it plays a role in stability and nuclear localization; however, its exact function is poorly understood. Two CRISPR-Cas9 generated *aip* mutant zebrafish (*Danio rerio*) lines were utilized. Homozygous mutants (*aip* ^*−/−*^) from either line died at 7–10-days post-fertilization (dpf), while no such effect is observed in *ahr* mutant lines. Larvae were exposed to four toxic AHR ligands from diverse chemical classes. *aip* ^*−/−*^ larvae exposed to PCB126, 5-nitroacenaphthene, and benzo(k) fluoranthene showed reduced sensitivity compared to both heterozygous (*aip* ^*+/−*^) and wildtype (*aip* ^*+/+*^) larvae. However, changes in sensitivity were ligand-dependent. In contrast, *aip* ^*−/−*^ larvae demonstrated increased sensitivity to leflunomide. mRNA sequencing was performed at five dpf on unexposed larvae of all genotypes from both mutant lines. Approximately 1,000 differentially expressed genes and alternative splicing products were observed in *aip* ^*−/−*^ larvae. Gene sets and transcription factor binding motifs involved in cellular stress, apoptosis, innate immune response, proteolysis, replication, and metabolism were enriched. Single-cell deconvolution of the bulk RNA-seq results was performed using MuSiC, which estimated transcriptomic contributions from 28 different cell types, with significant shifts in transcriptional abundance estimated for cell types from structural tissues of mesodermal origin, neural tissues, immune cells, and metabolic organs. Our findings highlight the context-dependent nature of Aip in Ahr modulation and the critical roles that Aip plays in regulating cellular homeostasis.

## 1 Introduction

The aryl hydrocarbon receptor (AHR) is a ligand-dependent transcription factor responsible for the expression of several xenobiotic metabolizing enzymes essential for adaptive responses to xenobiotics (Denison et al., 2011; Nukaya et al., 2010). Its ligand binding domain is capable of interaction with a broad range of xenobiotic compounds such as polycyclic aromatic hydrocarbons (PAHs), halogenated aromatic hydrocarbons (HAHs), and polychlorinated biphenyls (PCBs). TCDD (2,3,7,8-tetrachlorodibenzo-p-dioxin) is a particularly high affinity AHR ligand and the prototypical exogenous ligand of AHR (Poland and Kende, 1976). Additionally, natural products, pharmaceuticals, and endogenous compounds such as arachidonic acid, tryptophan metabolites, and heme metabolites have been described as AHR ligands (Avilla et al., 2020; Wen et al., 2023).

Zebrafish (*Danio rerio*) possess three *ahr* genes (*ahr1a*, *ahr1b*, and *ahr2*). *ahr1a* is orthologous to mammalian *AHR*. However, despite their evolutionary relationship, their exact functions may differ (Shankar et al., 2020; Hahn et al., 2017). *ahr2* is present in most tissues during developmental and adult stages, and is orthologous to *ahr2b* in other fish species (Karchner et al., 2005; Tanguay et al., 1999; Andreasen E. A. et al., 2002; Sugden et al., 2017). Expression of *ahr1a* and *ahr1b* is more restricted, largely localized to the liver and eye at 52 hpf, respectively (Sugden et al., 2017). Additionally, *ahr1b* knockdown was shown to reduce leflunomide-induced vascular Cyp1a expression at 120 hpf (Goodale et al., 2012). *ahr2* and *ahr1b* were shown to bind TCDD *in vitro*, while *ahr1a* was not (Prasch et al., 2006; Tanguay et al., 2000; Karchner et al., 2005; Andreasen E. et al., 2002). Further, TCDD exposure increased expression of *ahr2* and *ahr1a*, but not *ahr1b* (Andreasen E. et al., 2002; Andreasen E. A. et al., 2002; Garcia et al., 2018; Karchner et al., 2005; Tanguay et al., 1999; Ulin et al., 2019). *ahr2*-null zebrafish have decreased survival and diminished reproductive health, highlighting its endogenous roles. Endogenous roles for Ahr1a and Ahr1b have not been identified in zebrafish, and mutants of both are phenotypically normal (Shankar et al., 2020). These studies highlight the complexity of AHR regulation in zebrafish.

The AHR interacting protein (AIP) is also known as the X-associated protein-2 (XAP2), AH receptor-associated 9 (ARA9), or FK506-binding protein 37 (FKBP37). It belongs to a family of proteins that contain tetratricopeptide repeat (TRP) domains. AIP contains three TRP motifs and a C-terminal α-7 helix which mediate molecular interactions with other proteins (Trivellin and Korbonits, 2011).

The nonactivated AHR forms a complex with two heat shock protein 90 (HSP90) units, cochaperone p23, and AIP. Two molecules of HSP90 shape AHR’s ligand binding domain while negatively regulating AHR until ligand binding occurs. P23 interacts with the complex through HSP90 and is thought to help stabilize it and favor nuclear transport, but it is not required for AHR function (Trivellin and Korbonits, 2011; Cox and Miller, 2004; Flaveny et al., 2009). Interacting through its TRP domains, AIP binds both AHR and HSP90, stabilizing the complex and preventing proteasomal degradation of AHR (Kazzaz et al., 2024). Overexpression and co-expression studies demonstrated that AIP is important for keeping AHR in the cytoplasm rather than the nucleus (Petrulis et al., 2000). Further, overexpression of AIP has been shown to increase the sensitivity and the transcriptional response of AHR to dioxin (LaPres et al., 2000).

Upon ligand binding, AHR translocates to the nucleus and binds to ARNT. This active heterodimer binds xenobiotic responsive elements (XREs) activating transcription of phase I metabolizing enzymes such as cytochrome P450 one family (CYP1), and phase II enzymes including glutathione S-transferases (GST), UDP-glucuronosyltransferases (UGT), and NADPH quinone oxidoreductase 1 (NQO1) (Larigot et al., 2018; Yeager et al., 2009). While these enzymes often have a protective effect, they can also lead to the formation of toxic metabolites, like those seen following benzo [a]pyrene (BaP) exposure (Harrigan et al., 2004). The endogenous function of AHR is tissue-dependent, but it can play a role in mediating the cell cycle, cell proliferation, and immune system development (Avilla et al., 2020; Hao and Whitelaw, 2013; Denison et al., 2011).

Loss-of-function alleles of *Aip* are embryo-lethal in mice. *Aip*-null mice also experienced cardiac malformation, pericardial edema, decreased vasculature, and hemorrhaging (Lin et al., 2007; Kang et al., 2011). Additionally, knockout of *aip* ortholog *CG1847* in *Drosophila* is lethal during the larval stage (Aflorei et al., 2018) and *aip*-null *C. elegans* have severe phenotypes (Chen et al., 2017). In humans, *AIP* mutations are associated with familial isolated pituitary adenomas (FIPA) (Beckers et al., 2013; Daly et al., 2010). AHR loss-of-function does not produce such severe phenotypes. Beyond its role in AHR signaling, AIP interacts with a diverse range of proteins including nuclear receptors, transmembrane receptors, phosphodiesterases, mitochondrial proteins, and cytoskeletal regulators (Trivellin and Korbonits, 2011).

Several studies have investigated populations of Atlantic killifish (*Fundulus heteroclitus*) residing off the U.S. East Coast, finding that they had rapidly developed resistance to high levels of chemical pollutants (Nacci et al., 2010; Osterberg et al., 2018; Nacci et al., 2002; Reid et al., 2016; Nacci et al., 2010 trapped killifish at multiple highly contaminated sites and found that they had at least 500 times higher LC_20_ values when exposed to 3,3′,4,4′,5-pentachlorobiphenyl (PCB126) compared to killifish from sites with less contamination. The killifish embryonic EROD assay, which measures Cyp1a activity and can be used as a proxy for Ahr activation, demonstrated that fish from contaminated sites were less sensitive to Ahr activation when exposed to PCB126. Reid et al. (2016); Osterberg et al. (2018) performed whole genome sequencing (WGS) and RAD-seq, respectively, on resistant and sensitive killifish. They found that high levels of genetic diversity in killifish populations allowed rapid adaption. Further, sequence variation in multiple Ahr pathway genes were enriched in the analysis, and variants in Aip were some of the strongest candidates for resistance.

In this study we utilized two separate *aip*-mutant zebrafish lines (*aip* ^*wh86*^ and *aip* ^*wh239*^) to study the role of Aip in physiology and Ahr ligand-induced toxicity. Zebrafish have high fecundity and transparent embryos, making them ideal organisms for high-throughput physiological screening (Hill et al., 2005). Zebrafish also develop rapidly and have a much shorter generation time versus killifish (3 months vs. 12–24 months) making them an efficient model for rapid evaluation of chemical toxicity. Approximately 71% of human genes have at least one zebrafish ortholog (Howe et al., 2013). Importantly, AIP is evolutionarily conserved across species, making studies in zebrafish informative across taxa (Evans et al., 2008; Bell and Poland, 2000).

The *aip* ^*wh86*^ and *aip* ^*wh239*^ mutant lines were generated via a CRISPR-Cas9 induced deletion and early stop codon in exon two and exon five of *aip*, respectively (Karchner et al., 2026). As seen in *Aip*-null mice and *Drosophila*, zebrafish homozygous (*aip* ^*−/−*^ *)* for either mutation die at 7–10 dpf and no *aip* ^*−/−*^ adults were available for spawning. To overcome this, heterozygous (*aip* ^*+/−*^) adults were incrossed, resulting in offspring of unknown genotype in Mendelian ratios (1:2:1, *aip* ^*+/+*^ *: aip* ^*+/−*^ *: aip* ^*−/−*^) and requiring that each larva be genotyped following exposure or sample collection.

The role of Aip in the response to most Ahr ligands is not known. To investigate the role of Aip in chemical resistance, we exposed *aip* ^*wh239*^ mutant zebrafish to a diverse set of four known Ahr ligands, PCB126, 5-nitroacenaphthene (5-Nan), benzo(k)fluoranthene (BkF), and leflunomide, near their EC_50_ concentrations, the concertation which induced malformations in 50% of larvae at five dpf. Following exposure, individual larvae were assessed for morphological and behavioral defects at five dpf. Additionally, we investigated Aip’s role absent chemical exposure by performing mRNA sequencing on five dpf larvae from each genotype and line.

## 2 Materials and methods

### 2.1 *aip* ^*wh86*^ and *aip* ^*wh239*^ line development and genotyping

The generation of the *aip* ^*wh86*^ and *aip* ^*wh239*^ zebrafish lines using a CRISPR-Cas9 approach is described in Karchner et al. (2026). Briefly, the *aip* ^*wh86*^ line has a two bp deletion in exon two and encodes a 9.6kDa, 86 aa protein that lacks all of the TRP domains and the C-terminal α-helix. The *aip* ^*wh239*^ line has a five bp deletion in exon five and encodes a 38.3kDa, 342 aa protein that lacks two of three TRP domains and the C-terminal α-helix.

For genotyping these two lines, probe-based, custom TaqMan SNP Genotyping Assays (Applied Biosystems, Waltham, MA) were designed. This assay could be run in a 384-well format with the following master mix: 2.5 µL TaqMan Universal PCR Master Mix (Applied Biosystems, Waltham, MA), 0.25 µL 20X TaqMan assay, 1.5 µL ultrapure (UP) water, and 0.75 µL template genomic (gDNA) in a final reaction volume of 5 µL (Karchner et al., 2026). Guides RNAs, primers, and probes used are available in Supplementary Dataset 1-Table 1.

### 2.2 Fish husbandry

AIP mutant lines were generated in the Tüpfel long fin (TL) zebrafish line at Woods Hole Oceanographic Institution (Woods Hole, MA) and shipped to the Sinnhuber Aquatic Research Laboratory (SARL) at Oregon State University (Corvallis, OR) in 2021. There, the mutant TL lines were crossed with AB wildtype (WT) zebrafish and maintained following Institutional Animal Care and Use Committee protocols (IACUC-2024–0485 and IACUC-2024–0510).

Adults were kept in tanks with filtered, recirculating water supplemented with Instant Ocean salts (conductivity 1,000 µS, pH 7.4). Fish were kept in 6 L tanks at a density of approximately five fish/L tank at 28 °C under a 14/10 h light/dark cycle. Adult fish were fed GEMMA Micro 300 or 500 (Skretting, Inc., Fontaine les Vervins, France) twice a day. Larval and juvenile fish were fed GEMMA Micro 75 and 150, respectively, 3 times a day (Barton et al., 2016).

Pairwise spawning was performed by placing one male and one female fish in a spawning basket (Tecniplast S. p.A., Buguggiate, Italy) the afternoon before spawning. They were separated by a gate which was removed at 08:00 the morning of spawning. Alternatively, group spawning baskets were used to accommodate multiple males and females and were operated similarly. Pair spawning was used to generate new generations of fish and to track the number of contributing pairs, ensuring genetic diversity. Group spawns were used to generate offspring for experiments. Embryos were collected, staged, and maintained in embryo medium (EM) at 28 °C (Kimmel et al., 1995). The composition of the EM was as follows: 15 mM NaCl, 0.5mM KCl, 1 mM MgSO_4_, 0.15 mM KH_2_PO_4_, 0.05 mM Na_2_HPO_4_, and 0.7 mM NaHCO_3_ (Westerfield, 2007).

### 2.3 AHR ligand exposures

#### 2.3.1 AHR ligand range finding

To determine the EC_50_ concentration of each Ahr ligand, range finding was performed using *aip* ^*wh239+/+*^ larvae. At 4–5-h post-fertilization (hpf), embryos with chorions intact were plated into 96-well plates with 100 µL of EM and placed into an incubator at 28 °C. At six hpf an Ahr ligand was added using a HP D300e digital dispenser (Hewlett-Packard, Palo Alto, CA) (Truong et al., 2016). The following chemical stocks were used: 125 μg/mL PCB126, 10.14 mM 5-Nan, 10 mM BkF, and 10 mM leflunomide. All chemicals were dissolved in 100% dimethyl sulfoxide (DMSO). Range finding was performed with five concentrations of each chemical and a DMSO control. All wells were normalized to the highest concentration of DMSO (0.01%–0.6%). Concentrations, purity, CAS numbers, vendors, DMSO normalization, and sample size are available in Supplementary Dataset 1-Table 2.

Plates were sealed with ThermalSeal RTS silicone adhesive PCR film (Excel Scientific, Victorville, CA) and incubated at 28 °C under dark conditions. Plates were agitated on an orbital shaker at 235 RPM for the first 18 h (Truong et al., 2016). At five dpf larvae were screened for incidence of mortality and 11 different morphological endpoints including mortality as binary outcomes (Supplementary Dataset 1-Table 3). Morphological data were entered into our laboratory information management system, the Zebrafish Acquisition and Analysis Program (ZAAP). Dose-response modeling was performed as previously described and EC_50_ concentration was estimated for each chemical (Truong et al., 2014; Truong et al., 2016; Truong et al., 2022).

#### 2.3.2 EC_50_ AHR ligand exposure, morphological and behavioral assessment

For each chemical, we exposed 576 larvae at the predetermined EC_50_ concentration, rather than at multiple concentrations. Since *aip* ^*+/−*^ adults were used for spawning, all larvae were of unknown genotype, with each individual larvae requiring genotyping following exposure. Further, half of any clutch were *aip* ^*+/−*^ offspring, while only a quarter were *aip* ^*+/+*^ or *aip* ^*−/−*^ larvae, which effectively doubled the number of animals required to ensure sufficient statistical power. To increase throughput of experiments and reduce the unnecessary use of animals, exposures were performed at a single concentration.

The EC_50_ concentrations were determined to be 0.2 µM, 11 μM, 40 μM, and 0.75 µM for PCB126, 5-Nan, BkF, and leflunomide, respectively. Embryos were plated into six 96-well plates with a layout of half (n = 48) exposed to an Ahr ligand or vehicle control (0.0075%–0.4% DMSO) (Supplementary Dataset 1-Table 2). The DMSO concentration used for vehicle control wells was intended to normalize within experiment, so that chemical effects were compared against the same DMSO concentration. DMSO has previously been shown not to effect morphology at up to 1% and behavior at up to 0.55% in zebrafish developmental assays (Hoyberghs et al., 2021; Christou et al., 2020). Plates were incubated as described in Section 2.3.1 until five dpf when the larvae were assessed in the larval photomotor response (LPR) and larval startle response (LSR) assays, followed by morbidity and mortality assessment as described in Section 2.3.1.

The differences in the total distance traveled in LPR between exposed and control fish can elucidate impacts to brain and visual pathways (Dasgupta et al., 2022). The LSR assay immediately followed the LPR assay. Larvae were exposed to an intermittent 400 Hz auditory stimulus for 1 s, and their movement response was tracked for 9 s, and this cycle was repeated 10 times. Differences in movement between exposed and control fish can be used to assess impacts to neurobehavioral development (Dasgupta et al., 2020). The assay measured LSR total distance as an endpoint.

#### 2.3.3 96-Well larval genotyping following EC_50_ exposure

Genotyping was performed on 96-well plates following completion of 5dpf morphological and behavioral assessments: 50 µL of EM was removed from each well of the 96-well plate and 5 µL of 4 mg/mL tricaine was added to each well to euthanize the larvae. Images of each well were acquired using the Kestrel Multi-Camera Array Microscope (MCAM) (Ramona Optics Inc., Durham, NC). 10 min after the tricaine was added, 55 µL of 100 mM NaOH was introduced to each well making the final concentration of NaOH 50 mM. A multichannel pipette was used to move larvae to a 96-well PCR plate one column at a time. The plate was covered with an adhesive foil seal (VWR, Radnor, PA), placed in a thermocycler, and the larvae were digested at 100 °C for 15 min.

The plate was vortexed and centrifuged at 900x G for 3 min. A second 96-well PCR plate was prepared with 75 µL of UP H_2_O and 2.5 µL of 1 M Tris, pH 8.0 to neutralize. Using a multichannel pipette, 25 µL of homogenate was transferred to the neutralization plate. These plates were sealed with a foil seal, vortexed and centrifuged at 900x G for 1 min. Once completed for all six plates, the neutralized sample homogenates were genotyped in 384-well plates using the custom TaqMan SNP Genotyping Assay as described in Section 2.1.

#### 2.3.4 EC_50_ exposure statistical analysis

Morphological and behavioral data per well was exported from ZAAP as CSV files and the genotype of each replicate was added to the table. R version 4.4.1 (Team, 2021) was used to perform statistical analyses. Samples that could not be genotyped (often due to mortality) were removed prior to analysis.

For the morphological analysis, any larvae that were marked as “mortality” or had a well quality concern were excluded. A Kruskal–Wallis test, a non-parametric alternative to one-way analysis of variance (ANOVA), was used to test for a significant overall effect for endpoints available for a given chemical. If a significant overall effect was indicated (*p*-value ≤0.05), *post hoc* pairwise comparisons using Dunn’s test with Holm correction for multiple testing were used to evaluate genotype and concentration effects using predefined, biologically relevant comparisons of morphological endpoints (Supplementary Dataset 1-Table 4). Adjusted *p*-values ≤0.05 were considered statistically significant.

In all behavioral analyses, larvae with morphological defects were removed prior to analysis. Outliers were identified separately for each behavioral endpoint using the 1.5 • interquartile range (IQR) method. Values below Q1 – 1.5 • IQR and above Q3 + 1.5 • IQR were excluded from the analysis.

To test if baseline behavior varied by genotype without chemical exposure, behavioral data of unexposed larvae from the four EC_50_ AHR ligand exposures were assessed. To determine if movement varied by genotype and account for potential confounding due to experiments being performed on separate days, the following linear mixed model was fit: movement ∼ genotype + dataset + (1|dataset:plate). The model variables were as follows: “movement” is movement of larvae measured in mm, “genotype” is the genotype of the larvae, “dataset” adjusts for differences between the four independent experiments, and the interaction between “plate” within “dataset” accounts for plate effects within each independent experiment. Statistical significance of behavior endpoints was determined using type III ANOVA. Post-hoc pairwise comparisons were performed by assessing genotype differences using model-based estimated marginal means followed by pairwise t-tests for each comparison. Benjamini–Hochberg false discovery rate (FDR) adjusted *p*-values were presented with adjusted *p*-values ≤0.05 considered statistically significant. The LSR data required log transformation, altering the model to include log (1 + movement).

To assess whether AHR ligand exposure altered behavioral response in a genotype-dependent manner, the following linear mixed model was utilized: movement ∼ genotype • exposure + (1|plate). The model variables were as follows: “movement” is the distance traveled by larvae in the LPR assay (measured in mm), “genotype” is the genotype of the larvae, “exposure” is the chemical exposure status (exposed or control), and “plate” accounts for a potential plate effect within the experiment. Finally, the interaction between “genotype” and “exposure” tested whether there were any genotype-dependent behavioral responses due to exposure. The remaining statistical analysis was performed as described above. Concentrations and sample sizes can be found in Supplementary Dataset 1-Table 2.

### 2.4 Larval RNA sequencing

#### 2.4.1 Sample preparation

Healthy, six to eight hpf embryos with chorions intact were selected, plated into 96-well plates pre-filled with 100 µL of EM, and placed into an incubator at 28 °C. Two 96-well plates were set up for each line. At five dpf the plates were screened for mortality and malformed larvae, which were excluded from sampling.

50 µL of EM was removed from each well and the larvae were euthanized by placing the plates on ice for 10 min. The plates were removed from ice and 50 µL of RNA/DNA Shield (Zymo Research, Irvine, CA) was added to each well of a plate and left to rest for 30 min. Next, a multichannel pipette was used to dispense 100 µL of RNA lysis buffer (Zymo Research, Irvine, CA) to the first column of a plate which was immediately pipetted back up, attempting to remove the larvae with it. This solution was pipetted into a 96 round well 1.1 mL deep well homogenization plate (Axygen, Corning, NY) prefilled with 400 µL of 1.0 mm zirconium oxide beads (Next Advance Inc., Troy, NY) and 500 µL of RNA lysis buffer. The remaining 100 µL of solution in the plate was pipetted into the homogenization plate, removing any remaining larvae. This was repeated for all columns in the plate.

The homogenization plate was sealed with a silicone seal and homogenized using a Mini-Beadbeater-96 (BioSpec Products, Inc., Bartlesville, OK) for 1 min and 15 s at 2400 RPM. The homogenized plates were centrifuged for 5 min at 1700 G. A 96-well liquidator (Mettler Toledo, Greifensee, Switzerland) was used to transfer 550 µL of supernatant to a fresh KingFisher 96 deep-well plate (Thermo Fisher Scientific Inc., Waltham, MA). The sample plate was sealed with adhesive foil and stored at −20 °C until further processing.

#### 2.4.2 Sample genotyping from RNA lysis buffer

Since *aip* ^*+/−*^ adults were incrossed, all the samples needed to be genotyped. The Zymo RNA lysis buffer is a harsh denaturing solution that does not allow the enzymatic activity necessary for PCR to take place. PCR of the homogenized samples was performed by diluting a small portion of the homogenate in UP water as follows: 10 µL of supernatant was removed from the homogenization plate and added to a 96-well PCR plate. 1 µL of this homogenate was moved to a separate 96-well PCR plate containing 49 µL of UP water. This 1:49, buffer:H_2_O solution was diluted enough to permit PCR yet contained enough gDNA for probe-based genotyping using the previously described custom TaqMan SNP Genotyping Assay (Section 2.1).

#### 2.4.3 Total RNA extraction

After the genotype of each sample was determined, the sample plates were thawed and 32 larvae from each genotype and line were selected for Total RNA extraction. A KingFisher Apex liquid handler system (Thermo Fisher Scientific Inc., Waltham, MA) and a Quick-RNA MagBead kit (Zymo Research, Irvine, CA) were used to perform the extractions in a 96-well format. The Zymo kit requires an equal volume of 100% ethanol to be mixed with the sample for extraction but has a max volume of 1,000 µL. Since 550 µL of supernatant was collected, the final volume with ethanol was 1,100 µL and had to be split between two extraction plates. Each sample was individually moved and equally divided between two extraction plates. The well position of the original sample plate was tracked throughout this process and into the sample submission. 275 μL of ethanol and 30 µL of mag beads were added using a multichannel pipette and the extraction was carried out following the kit protocol including a DNase I digestion step. Briefly, the solution was mixed and RNA bound for 10 min, washed twice in 500 µL of DNA/RNA wash for 2 min each, then washed twice in 500 µL of ethanol for 2 min each. DNase treatment was performed for 10 min using 50 µL of DNase digestion buffer and RNA was rebound using 500 µL of RNA prep buffer for 10 min. RNA was washed twice in 500 µL of ethanol for 2 min each, dried for 10 min, and eluted into 50 µL of UP water by mixing for 9 min.

Individual larval RNA samples were pooled following RNA extraction to obtain sufficient RNA input and to average individual variability within each replicate, reducing inter-replicate variation. Each pooled replicate was created by combining five individual samples. Samples with the highest concentration and RIN scores >8 were selected to be pooled. The average concentrations of the pooled RNA samples were 9.16, 7.99, and 7.35 ng/μL for *aip* ^*+/+*^, *aip* ^*+/−*^, and *aip* ^*−/−*^, respectively. The RIN and concentration of each sample was determined using an Agilent 4,150 TapeStation System (Agilent, Santa Clara, CA) and a qubit fluorometer (Invitrogen, Waltham, MA), respectively. Sample concentration and quality was further characterized by Lexogen GmbH (Vienna, Austria) following sample submission. There, average concentrations (Nanodrop 2000c UV-Vis spectrophotometry) and RNA quality number (RQN) (Agilent Fragment Analyzer System) of the pooled RNA samples were measured as 13.99, 11.55, and 11.20 ng/μL and 9.64, 9.67, and 9.66 respectively, for *aip* ^*+/+*^, *aip* ^*+/−*^, and *aip* ^*−/−*^ samples.

#### 2.4.4 Lexogen sequencing of RNA samples

Pooled total RNA samples were submitted to Lexogen for sequencing. Four replicates were sequenced per genotype and line (four *aip* ^*+/+*^, *aip* ^*+/−*^, and *aip* ^*−/−*^ pools for both *aip* ^*wh86*^ and *aip* ^*wh239*^ lines) totaling 24 samples. Library preparation was performed using the CORALL mRNA-Seq V2 whole transcriptome sequencing kit (Lexogen GmbH, Vienna, Austria). 150bp paired end reads were sequenced on an AVITI sequencing platform (Element Biosciences, San Diego, California).

#### 2.4.5 RNA-seq data processing

Initial data processing and analysis was performed by Lexogen. It included trimming adapter sequences, primers, and poly-A tails as well as quality trimming and filtering using cutadapt tool (Martin, 2011) The median number of filtered reads was 30.82 million, with the minimum being 27.85 million, and the maximum being 33.51 million. The Star aligner (Dobin et al., 2013) was used to align reads to the GRCz11 genome assembly. The median percentage of uniquely mapped reads was 85.4%, with the minimum being 83.7% and the maximum being 86.7%. Unique molecular identifier (UMI) tags were included in the samples and used to remove PCR duplicates. Reads with the same UMI that mapped to the same location were collapsed to one read. Following UMI collapsing, the median percentage of remaining reads was 58.9% with the minimum being 54.6% and the maximum being 66.1%. Transcript- and gene-level count matrices were generated which included 53,466 and 32,171 features, respectively. Raw and processed RNA-seq data are deposited in the NCBI Gene Expression Omnibus database (GEO accession #GSE328610). Transcript- and gene-level count matrices are also available in Supplementary Dataset 3-Tables 1, 2, respectively.

#### 2.4.6 DEG analysis of RNA-Seq data

DEG analysis was performed in R using the DESeq2 package (Love et al., 2014). The *aip* ^*wh86*^ and *aip* ^*wh239*^ lines were analyzed as separate datasets. Transcript IDs were mapped to Ensembl gene IDs (Dyer et al., 2025) and gene symbols via biomaRt (Durinck et al., 2005; Durinck et al., 2009) using the Ensembl *D. rerio* GRCz11.115 genome assembly. Transcripts counts that belonged to the same Ensembl gene ID were summed yielding a gene level count matrix. Transcripts that could not be mapped were discarded. Genes with counts <10 summed across all samples were excluded from further analysis.

The model counts ∼ genotype was fit using the DESeq () function and the following specific genotype-within-line comparisons were made: *aip* ^*wh86−/−*^ vs. *aip* ^*wh86+/+*^, *aip* ^*wh86+/−*^ vs. *aip* ^*wh86+/+*^, *aip* ^*wh239−/−*^ vs. *aip* ^*wh239+/+*^, and *aip* ^*wh239+/−*^ vs. *aip* ^*wh239+/+*^. An analysis was also performed for line-within-genotype comparisons: *aip* ^*wh239+/+*^ vs. *aip* ^*wh86+/+*^, *aip* ^*wh239+/−*^ vs. *aip* ^*wh86+/−*^, and *aip* ^*wh239−/−*^ vs. *aip* ^*wh86−/−*^.

This returned log_2_ (fold change) (L2FC) values, wald test *p*-values, and Benjamini–Hochberg FDR-adjusted *p*-value (*p*adj). Additionally, the apeglm method (Zhu et al., 2019) for the lfcShrink () function in DESeq2 was used to shrink L2FC values to obtain more stable size estimates for low-count genes. This resulted in DEG tables with 24,510, 24,399, and 25,269 genes for the *aip* ^*wh86*^, *aip* ^*wh239*^, and line-within-genotype datasets, respectively. Transcripts were considered DEGs if they had a *p*adj ≤ 0.05 and a shrunken L2FC ≥ |1|.

Principal component analysis (PCA) was performed on gene expression values generated by DESeq2 following transformation using the variance-stabilizing transformation (VST) of the 500 most variable genes across samples. A sample-sample distance heatmap was generated using Euclidean distances computed from the VST counts using the pheatmap package (Kolde, 2025). Samples were grouped using hierarchical clustering. Potential outlier samples were assessed using the PCA, sample-sample distance heatmap, and Cook’s distances. All samples were retained. Mean-variance (dispersion) relationship and MA plots were generated using DESeq2 and showed the expected patterns for RNA-seq data.

#### 2.4.7 Maternal aip transcript annotation

Since *aip* ^*+/−*^ adults were used to generate all offspring, maternal WT transcripts may have been present in *aip* ^*−/−*^ offspring due to WT mRNA carryover from the egg. This observation was confirmed in Karchner et al. (2026). Additionally, they observed that mutant transcripts in *aip* ^*+/−*^ and *aip* ^*−/−*^ larvae are expressed at very low levels, likely due to nonsense-mediated decay (NMD). We performed a similar analysis with the present data (Karchner et al., 2026).

Samtools (samtools index) (Danecek et al., 2021) was used to index the bam alignment file corresponding to each sample, generating bam index files (bai). The bam and bai files were loaded into the integrated genome viewer web app (IGV.org/app) (Robinson et al., 2011). IGV was used to visualize and count both WT and mutant transcripts for each individual sample in both lines and all genotypes.

The quantified transcript data was analyzed in R. Nonparametric Kruskal–Wallis tests were used to assess differences in transcript abundance across genotype-line groups. Dunn’s *post hoc* tests with Holm correction were used to evaluate predefined genotype-within-line and line-within-genotype biologically relevant comparisons. Adjusted *p*-values ≤0.05 were considered statistically significant.

#### 2.4.8 GSEA network analysis of RNA-Seq data

Gene set enrichment analysis (GSEA) was performed on the RNA-seq data to investigate biological pathways perturbed by *aip* mutation in both lines. For this analysis Kyoto Encyclopedia of Genes and Genomes (KEGG) (Kanehisa and Goto, 2000) and Gene Ontology: Biological Process (GO:BP) (Ashburner et al., 2000; Gene Ontology, 2026) terms were used. Using the biomaRt and KEGGREST (Tenenbaum, 2025) packages, GO:BP and KEGG gene sets were assembled for GSEA. Annotations were retrieved and exported as a GMT file with 2,159 gene sets. As previously described for the DEG analysis, DESeq2 was used to generate an *aip* ^*−/−*^ vs. *aip* ^*+/+*^ DESeq2 object for each line. Genes were ranked using Wald test statistic from the DESeq2 object “stat” ( $stat=\log_{2}\left(FC\right)/SE$ ) and exported as a RNK file.

The GSEA software (Subramanian et al., 2005; Mootha et al., 2003) was used to perform the analysis. Both lines were analyzed separately. The GMT and RNK file were loaded and GSEA pre-ranked analysis was performed using 1,000 permutations, no collapsing, and weighted enrichment. Gene sets with less than 15 genes and more than 250 genes were excluded, leaving 318 gene sets in the analysis. Filtering removed a large number of small gene sets which protected statistical power following multiple testing, and avoided large, biologically nonspecific terms.

Once the GSEA was complete, Cytoscape (Shannon et al., 2003) with the Enrichment Map (Reimand et al., 2019) and yFiles Layout Algorithms (Wiese et al., 2004) apps were used to generate the network. The positive and negative enrichment files and the rank file from the GSEA output were loaded into Enrichment Map. Using advanced options the FDR *q*-value cutoff was set to 0.05, data set edges was set to combine across data sets, cutoff metric was set to overlap, and cutoff was set to 0.25. A cutoff of 0.25 meant that at least 25% of the genes in the smaller gene set must overlap with the larger gene set for them to connect with an edge. This was performed for both lines and an overlapping gene set network was generated.

The layout was set to radial using the yFiles layout app. The nodes (gene sets) were colored by dataset, the shape was determined by ontology type (KEGG or GO:BP), node size was determined by gene set size, connection edge size was determined by number of shared genes, and the outline color was determined by normalized enrichment score (NES) direction (positive or negative). Positive NES meant that genes in a gene set were overall overexpressed in *aip* ^*−/−*^ larvae compared to *aip* ^*+/+*^ samples while a negative NES meant that a gene set’s representative genes were overall underexpressed. Nodes which did not have any connections to other nodes were removed from the network and represented in a heatmap which used a NES • -log_10_(FDR) scale. The network was further annotated in Illustrator (Adobe Inc., San Jose, CA) with ellipses drawn around groups of three or more similar nodes.

#### 2.4.9 Single cell deconvolution of bulk RNA-Seq data

Single cell deconvolution estimates cell-type-specific compositions from bulk RNA-seq data using a single-cell RNA-seq atlas from the same organism and developmental timepoint as a reference. This was used to highlight potential differences in transcriptional profile within specific cell types. For this analysis we utilized the MuSiC R package (Wang et al., 2019).

A five dpf zebrafish single-cell RNA-seq atlas was downloaded as a .h5ad file from zebrahub.sf.czbiohub.org (Lange et al., 2024), a publicly available resource for zebrafish single-cell atlases. The atlas was imported into R using the zellkonverter package (Zappia and Lun, 2025). The DelayedArray (Pagès, 2025a) and HDF5Array (Pagès, 2025b) packages were used to handle the large dataset and obtain raw count data. Cell types represented by fewer than two biological samples in the atlas were excluded from downstream analysis.

Raw single-cell counts were normalized to count per million (CPM) by dividing each cell’s counts by its library size and multiplying by 10^6^. Genes with non-zero variance across cell types were retained and the top 4,000 most variable genes were selected for deconvolution. Variance-based filtering was performed by computing mean gene expression for each cell type across all included cell types. Bulk RNA-seq data was loaded as a gene-level count matrix. The *aip* ^*wh86*^ and *aip* ^*wh239*^ lines were analyzed as separate data sets. Only *aip* ^*+/+*^ and *aip* ^*−/−*^ samples were retained for analysis. Raw counts were normalized to CPM using sample-specific library size. Genes with zero total counts were excluded from further analysis.

Cell-type proportion estimates were generated from the normalized bulk RNA-seq data and the single-cell reference using MuSiC. Estimated cell-type proportions and standard errors were exported as CSV files. Global differences in cell-type proportions between *aip* ^*+/+*^ and *aip* ^*−/−*^ for both lines were assessed using permutational multivariate analysis of variance (PERMANOVA), a non-parametric multivariate test analogous to ANOVA. If the global test was significant, two-sided Wilcoxon rank sum tests were performed to identify cell types contributing to the compositional differences between genotypes. Raw *p*-values ≤0.05 were considered significant and raw *p*-values ≤0.1 with effect sizes ≥7 were considered to be trending significant.

#### 2.4.10 Transcription factor binding motif analysis

To investigate potential common transcriptional regulators, a binding motif analysis was performed. The *aip* ^*wh86*^ and *aip* ^*wh239*^ datasets were analyzed separately. DEG tables for the *aip* ^*−/−*^ vs. *aip* ^*+/+*^ comparisons were loaded into R as TSV files and filtered, only retaining DEGs (*p*adj ≤ 0.05, shrunken L2FC ≥ |1|). Additionally, the gene-level count matrixes for each line were loaded into R as a TSV files. The gene count matrix serves as reference for background gene expression and only measurably expressed genes with read counts ≥10 in at least two samples were retained for the analysis.

The rtracklayer package (Lawrence et al., 2009) was used to import gene annotations from the Ensembl *D. rerio* GRCz11.115 genome assembly. Promoter sequences were defined as the genomic regions ±1 kb around the transcription start site using the GenomicRanges (Lawrence et al., 2013) and GenomeInfoDb (Arora et al., 2025) packages. The analyses were further divided into higher abundance DEGs or lower abundance DEGs for the aip^−/−^ vs. *aip* ^*+/+*^ comparison. Promoter sequences were extracted for each gene and written as FASTA files. Additionally, promoter sequences were extracted for all expressed genes using the previously mentioned gene count matrix and written as FASTA files.

The Meme Suite (Bailey et al., 2015) Analysis of Motif Enrichment (AME) tool (McLeay and Bailey, 2010) was used to perform motif enrichment. Promoter sequences from DEGs were compared against promoter sequences from the expressed background genes using the total-hits scoring method against the JASPAR 2026 vertebrate transcription factor (TF) motif database (Ovek Baydar et al., 2026). Statistical analysis was performed within AME using Fisher’s exact test with a Bonferroni correction. An adjusted *p*-value ≤0.05 was considered significant. Output files included enriched TF binding motif sequences, motif names/IDs, motif family/class, and adjusted *p*-values.

#### 2.4.11 Isoform-level differential transcript usage

In addition to DEG analysis, we utilized the transcript-level RNA-seq data to investigate differential transcript usage (DTU). DTU analysis is used to look at a gene’s alternative splicing products by testing for changes in proportion of transcript usage between conditions, while considering the overall change in expression of the corresponding gene. This differs from other analyses like differential transcript expression (DTE) which treats a transcript’s change in expression as an independent event without considering the gene’s overall change in expression (Froussios et al., 2019; Tekath and Dugas, 2021).

RNA-seq data was loaded into R as a transcript-level count matrix with the *aip* ^*wh86*^ and *aip* ^*wh239*^ lines analyzed as separate data sets. Only *aip* ^*+/+*^ and *aip* ^*−/−*^ samples were retained for analysis. Transcript IDs were linked to Ensembl gene IDs and gene symbols using biomaRt to import the Ensembl *D*. *rerio* GRCz11.115 genome assembly. The IsoformSwitchAnalyzeR package (Vitting-Seerup and Sandelin, 2017; Vitting-Seerup and Sandelin, 2019) was used to integrate the transcript counts and experimental design (aip^+/+^ was specified as the reference level) with the Ensembl *D. rerio* GRCz11.115 genome assembly producing one IsoformSwitchAnalyzeR object per *aip* mutant line.

Low confidence features (≤5 counts, average isoform fraction (IF) ≤ 1%|, genes with a single isoform) were removed. DTU analysis between *aip* ^*−/−*^ and *aip* ^*+/+*^ was performed using DEXSeq (Anders et al., 2012; Reyes et al., 2013). Transcripts were considered DTUs if they had Benjamini–Hochberg FDR-adjusted isoform switch *q*-values ≤0.05. DTUs were retained for downstream analysis if they had a change in isoform fraction (IF_aip_^+/+^ – IF_aip_^−/−^ = dIF) ≥ |0.1|.

Open reading frames (ORFs) were pulled from the reference genome and added to the IsoformSwitchAnalyzeR object for protein-level analysis. Nucleotide and amino acid sequences were extracted for transcripts with dIF ≥ |0.1|, and protein domains were annotated using Pfam (Blum et al., 2025; Paysan-Lafosse et al., 2025). Amino acid sequences were also used as an input for the following external protein feature prediction tools: CPC 2.0 (Kang et al., 2017; Kong et al., 2007) to assess coding potential of transcripts, SignalP 6.0 to predict signal peptides (Teufel et al., 2022) DeepTMHMM 1.0 (Hallgren et al., 2022) to predict transmembrane helices, DeepLoc2 (Odum et al., 2024) to predict subcellular localization, AIUPred (Erdos and Dosztanyi, 2024; Erdos et al., 2025) to predict intrinsically disordered regions, NetSurfP 3.0 (Hoie et al., 2022) to predict surface accessibility and secondary structure, and TargetP2 (Almagro Armenteros et al., 2019) to predict mitochondrial targeting peptides. Finally, intron retention was quantified using IsoformSwitchAnalyzeR.

IsoformSwitchAnalyzeR was then used to assess functional consequences of alternative splicing products including changes in coding potential, protein domains, signal peptides, transmembrane topology, subcellular localization, intrinsically disordered regions, mitochondrial targeting peptides, and intron retention. Only significant consequences were retained (*q* ≤ 0.05, dIF ≥ |0.1|).

## 3 Results

### 3.1 *aip* mutation alters susceptibility to ahr ligands

At five dpf, Aip mutants exposed to the vehicle control, DMSO, did not exhibit morphological differences between the genotypes, i.e., unexposed *aip* ^*wh239+/+*^, *aip* ^*wh239+/−*^ and *aip* ^*wh239−/−*^. Most statistically significant endpoints correlated with an increased incidence of the observed phenotype following chemical exposure in at least one genotype. Further, for many endpoints there was a significant difference in the incidence of morphological defects between the genotypes in the exposed group, demonstrating potential differential susceptibility. Results of the statistical analysis can be found in Supplementary Dataset 2-Table 2 and raw data is available in Supplementary Dataset 2-Table 1.

**FIGURE 1:**
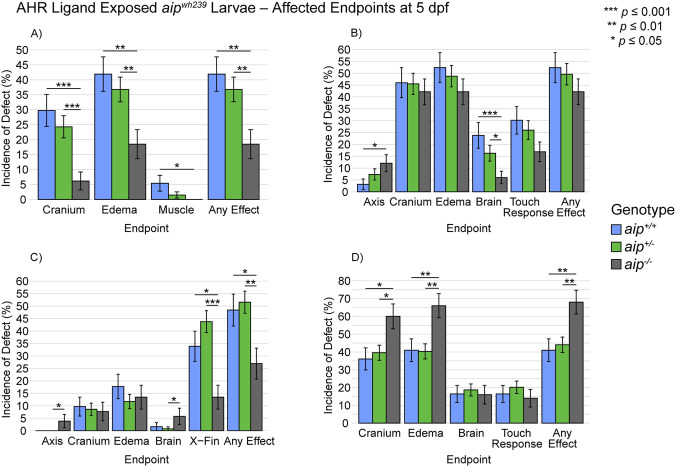
Morphological endpoints with significantly increased incidence in five dpf *aip* ^*wh239*^ larvae following exposure to Ahr ligands near their EC_50_ concentrations **(A)** 0.2 µM PCB126 **(B)** 11 µM 5-NAN **(C)** 40 μM BkF, and **(D)** 0.75 µM leflunomide. Affected endpoints are displayed on the x-axis, while the percent incidence of the endpoint is displayed on the y-axis. Significant differences between each genotype are denoted by an asterisk, * (*p*-value ≤0.05, Kruskal–Wallis test followed by Dunn’s *post hoc* tests with Holm correction, error bars = standard error). Each experiment demonstrated that *aip* ^*wh239−/−*^ larvae had differential susceptibility to Ahr ligands, with reduced sensitivity to all exposures except leflunomide, for which sensitivity was greater.

All three genotypes were exposed to a single concentration of each AHR ligand where 50% of *aip* ^*wh239+/+*^ larvae exhibited an effect (EC_50_). For each exposure, 11 morphological endpoints were assessed and a summative endpoint “any effect” was computed. Overall, *aip* ^*wh239−/−*^ larvae showed reduced sensitivity to PCB126 (Figure 1A). At 0.2 µM of PCB126, there was a significant difference in cranium, edema, muscle, and any effect phenotype between for *aip* ^*wh239−/−*^ and *aip* ^*wh239+/+*^ larvae. All the endpoints except for “muscle” were significantly different between *aip* ^*wh239−/−*^ and *aip* ^*wh239+/−*^.

Exposure to 11 µM 5-Nan caused significant changes in six endpoints: axis, cranium, edema, brain, touch response, and any effect endpoints, but only the axis and brain endpoints showed a significant difference between two genotypes (Figure 1B). *aip* ^*wh239−/−*^ exposed larvae had reduced incidence of the brain endpoint compared to both *aip* ^*wh239+/+*^ and *aip* ^*wh239+/−*^ larvae. However, *aip* ^*wh239−/−*^ larvae had increased incidence of the axis endpoint compared to *aip* ^*wh239+/+*^ larvae.

Exposure to 40 µM BkF also caused significant changes in six endpoints: axis, cranium, edema, brain, X-fin, and any effect endpoints (Figure 1C). In the exposure group, *aip* ^*wh239−/−*^ larvae had significantly reduced X-fin malformations compared to the other genotypes. Incidence of the cranium and edema endpoints were not significantly changed among the exposed genotypes. *aip* ^*wh239−/−*^ larvae had significantly greater incidence of both axis and brain endpoints compared to *aip* ^*wh239+/−*^ animals. However, any effect was still significantly lower in *aip* ^*wh239−/−*^ larvae, driven by reduced susceptibility to the X-fin phenotype.

In contrast to the first three exposures, when exposed to 0.75 µM leflunomide, *aip* ^*wh239−/−*^ zebrafish demonstrated increased sensitivity (Figure 1D). Cranium, edema, brain, touch response, and any effect endpoints had significant effects. *aip* ^*wh239−/−*^ animals had greater incidence of cranium, edema, and any effect endpoints, while the incidence of the brain and touch response endpoints were unchanged across the exposed genotypes.

### 3.2 Unexposed and exposed *aip* ^*wh239−/−*^ larvae have an altered photomotor and startle response

**FIGURE 2:**
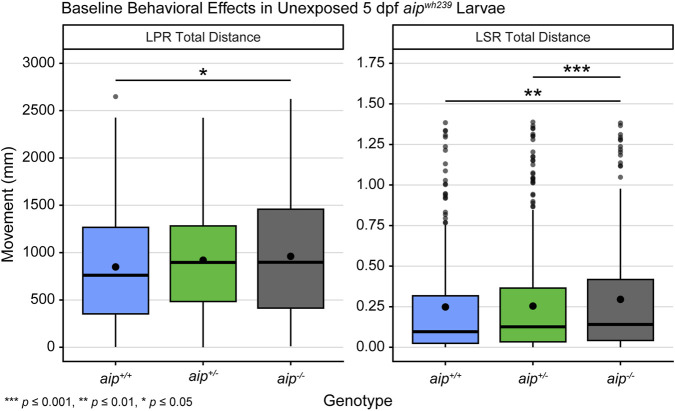
Behavioral analysis of DMSO-exposed five dpf larvae from the *aip* ^*wh239*^ line. Data is from control larvae for each of the four EC_50_ Ahr ligand exposures. A linear mixed model was fit, including “dataset” as a variable to account for experiment-to-experiment variation. Genotype is presented on the x-axis, and larval movement in mm is presented on the y-axis for LPR and LSR total distance. Boxplots show the median (horizontal line), mean (circle), interquartile range (box), and 1.5x the interquartile range (whiskers). Significant differences between each genotype are denoted by an asterisk, * (*p*-value ≤0.05, type III ANOVA followed by pairwise t-tests of model-based estimated marginal means adjusted using Benjamini–Hochberg FDR correction). Unexposed *aip* ^*wh239−/−*^ larvae had significantly increased movement in the LPR and LSR assays when compared to *aip* ^*wh239+/+*^ larvae.

Unexposed *aip* ^*wh239−/−*^ larvae travelled significantly greater distance (hyperactivity) than *aip* ^*wh239+/+*^ larvae during the light-dark transition of the LPR assay. Specifically, *aip* ^*wh239−/−*^ larvae were hyperactive during the light phase of the assay. Larval movement is typically low in the light phase and higher in the dark phase. Additionally, the total distance travelled after an acoustic vibration (larval startle response (LSR)) was significantly greater in *aip* ^*wh239−/−*^ larvae compared to the other genotypes. There was no difference between *aip* ^*wh239+/+*^ and *aip* ^*wh239+/−*^ animals in the LPR assay (Figure 2). Estimates and *p*-values of fixed effects and pairwise comparisons for baseline behavior can be found in Supplementary Dataset 2-Table 3.

When investigating PCB126 exposure effects within genotype it was determined that PCB126-exposed *aip* ^*wh239+/+*^ and *aip* ^*wh239+/−*^ larvae were hypoactive in the LPR assay. These differences were driven by changes in movement during the light phase of the assay. *aip* ^*wh239−/−*^ larval photomotor behavior did not differ between control and exposed. The *aip* ^*wh239+/+*^ or *aip* ^*wh239+/−*^ vs. *aip* ^*wh239−/−*^ genotype • exposure interactions did not have significant FDR-adjusted *p*-values (≤0.05), indicating that PCB126 exposure did not produce greater hypoactivity in any of the genotypes. While the within genotype effects suggest that homozygous *aip* mutation may be protective against PCB126 behavioral effects, the interaction effects were nonsignificant and homozygous *aip* mutation cannot be definitively linked to altered behavioral sensitivity (Supplementary Dataset 2-Table 4).

All 5-Nan exposed larvae, regardless of genotype, were hypoactive during the LPR assay, and *aip* ^*wh239+/−*^ larvae were hypoactive in the LSR assay. Examination of the genotype • exposure interaction for LPR total distance indicated that 5-Nan exposure led to greater hypoactivity in *aip* ^*wh239+/−*^ larvae compared to *aip* ^*wh239−/−*^ and *aip* ^*wh239+/+*^ larvae. While the difference in 5-Nan-induced hypoactivity between *aip* ^*wh239+/−*^ and *aip* ^*wh239−/−*^ larvae suggests that homozygous *aip* mutation may be protective against 5-Nan exposure, the same behavioral alteration was observed between *aip* ^*wh239+/−*^ and *aip* ^*wh239+/+*^ larvae, reducing the certainty that this behavioral phenotype was induced by *aip* mutation. These changes were driven by decreased movement in the dark phase of the LPR assay (Supplementary Dataset 2-Table 4).

The BkF within genotype exposure effects showed that all exposed genotypes were hypoactive in both the light and dark phases of the LPR assay, but hyperactive in the LSR assay. However, none of the genotype • exposure interactions had significant FDR-adjusted *p*-values, suggesting that *aip* mutation does not protect against BkF-induced behavioral alterations (Supplementary Dataset 2-Table 4).

**FIGURE 3:**
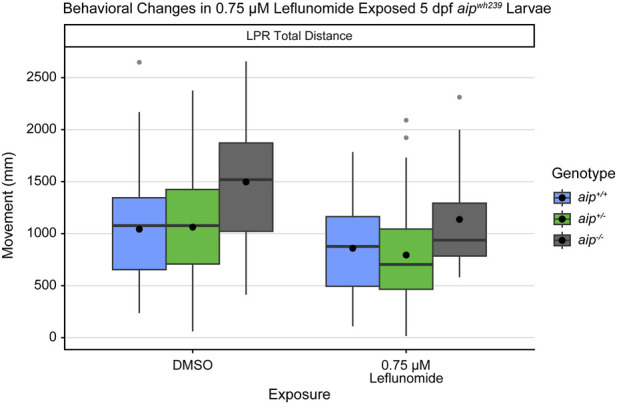
Behavioral analysis of leflunomide-exposed five dpf larvae from the *aip* ^*wh239*^ line. Exposure condition is presented on the x-axis, and larval movement in mm is presented on the y-axis for LPR total distance. Genotype is denoted by color. Boxplots show the median (horizontal line), mean (circle), interquartile range (box), and 1.5x the interquartile range (whiskers). When exposed to leflunomide, the reduction in movement (hypoactivity) of *aip* ^*wh239−/−*^ larvae was significantly greater compared to the other genotypes (*p*-value ≤0.05, type III ANOVA followed by pairwise t-tests of model-based estimated marginal means adjusted using Benjamini–Hochberg FDR correction).

As with the BkF exposure, the leflunomide exposure within genotype analysis showed that all exposed genotypes were hypoactive in both the light and dark phases of the LPR assay, but hyperactive in the LSR assay. Leflunomide exposure also induced the clearest genotype-dependent behavioral effects. The genotype • exposure interaction showed that *aip* ^*wh239−/−*^ larvae had greater hypoactivity during the light phases of the LPR assay due to leflunomide exposure than did the other genotypes (Figure 3). This suggests that *aip* mutation resulted in greater sensitivity to leflunomide-induced behavioral alterations. Estimates and *p*-values of fixed effects and pairwise comparisons for chemical exposures can be found in Supplementary Dataset 2-Table 4. Raw data is available in Supplementary Dataset 2-Table 1.

### 3.3 Homozygous *aip* mutation drives extensive transcriptomic changes in larvae

**FIGURE 4:**
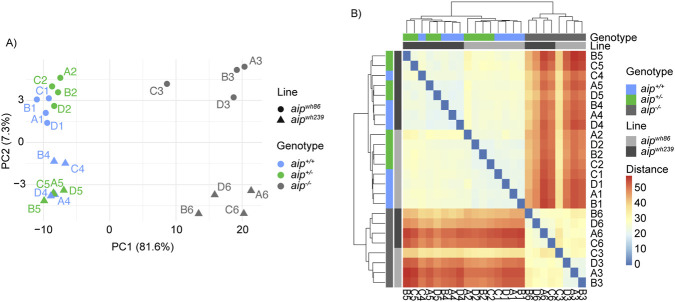
PCA plot **(A)** and sample–sample distance heatmap **(B)** of all 24 five dpf RNA-seq samples with sample ID. PCA was performed on the 500 most variable genes across the samples using variance-stabilizing transformed (VST) counts. The sample-sample distance heatmap was generated using Euclidean distances computed from the VST counts and grouped via hierarchical clustering. Both plots show the different lines and, to a greater extent, the *aip* ^*−/−*^ samples cluster separately.

To investigate transcriptional changes, four pools of five five dpf larvae were sequenced from each line and genotype. To observe transcriptomic variance between each genotype a PCA (Figure 4) and DEG analysis (Table 1) were performed. *aip* ^+/+^ and *aip* ^+/−^ samples distinctly separated from *aip* ^−/−^ samples along PC1 which contained a large amount of the observed variance (81.6%) (Figure 4A). The sample-sample distance heatmap (Figure 4B) also estimated a large difference between *aip* ^−/−^ larvae and the other genotypes. When comparing *aip* ^*wh86*−/−^ vs. *aip* ^*wh86*+/+^ samples 1,158 DEGs (*p*adj ≤ 0.05, shrunken L2FC ≥ |1|) were found, while 927 DEGs were found in an *aip* ^*wh239*−/−^ vs. *aip* ^*wh239*+/+^ comparison. 658 DEGs overlapped between the lines while 500 and 269 DEGS were unique to the *aip* ^*wh86*^ and *aip* ^*wh239*^ lines, respectively. Only one DEG that was present in both datasets differed in abundance direction; *si:dkey-22i16.9* was in greater abundance in the *aip* ^*wh86*^ dataset but lower in the *aip* ^*wh239*^ dataset. When comparing *aip* ^+/−^ vs. *aip* ^+/+^ there were six and 4 DEGs found for the *aip* ^*wh86*^ and *aip* ^*wh239*^ lines, respectively. This is consistent with the high degree of similarity observed between *aip* ^+/−^ and *aip* ^+/+^ animals (Karchner et al., 2026).

**TABLE 1:** DEG (*p*adj ≤ 0.05, shrunken L2FC ≥ |1|) counts for the five dpf RNA-seq data. Each comparison performed using DESeq2 is shown and includes higher abundance, lower abundance, and the total number of DEGS. *aip* ^*−/−*^ larvae had a high number of DEGs compared to *aip* ^*+/+*^ larvae, where *aip* ^*+/−*^ animals had very few. There was a moderate number of DEGs when comparing between the lines but within genotype. The *aip* ^*wh239+/+*^ vs. *aip* ^*wh86+/+*^ comparison revealed 105 DEGs in total, suggesting some divergence between the lines, even in WT animals. This is supported by the PCA and heatmap results in Figure 4.

Group 1 vs.	Group 2	DEG up	DEG down	DEG total
*aip* ^*wh86−/−*^	*aip* ^*wh86+/+*^	639	519	1,158
*aip* ^*wh86+/−*^	*aip* ^*wh86+/+*^	6	0	6
*aip* ^*wh239−/−*^	*aip* ^*wh239+/+*^	559	368	927
*aip* ^*wh239+/−*^	*aip* ^*wh239+/+*^	4	0	4
*aip* ^*wh239−/−*^	*aip* ^*wh86−/−*^	93	82	175
*aip* ^*wh239+/+*^	*aip* ^*wh86+/+*^	39	66	105
*aip* ^*wh239+/−*^	*aip* ^*wh86+/−*^	47	81	128

The *aip* ^*wh86*^ and *aip* ^*wh239*^ lines separated along PC2 with much less variance (7.3%), including a separation of *aip* ^+/+^ and *aip* ^+/−^ samples between lines and a slightly greater separation between *aip* ^−/−^ samples (Figure 4A). There is a degree of difference between *aip* ^+/+^ samples in either line with 105 DEGs found. 128 DEGs were found when comparing *aip* ^*+/−*^ samples and 175 DEGs when comparing *aip* ^*−/−*^ samples from the different lines. The variance between *aip* ^+/+^ samples might be explained by the fact that the *aip* ^*wh86*^ and *aip* ^*wh239*^ lines originated from independent founders, and later from separate breeding stocks (Karchner et al., 2026). This divergence could complicate further comparison between lines. DEG tables are available in Supplementary Dataset 4.

### 3.4 Homozygous mutants have few *aip* transcripts

*aip* ^*wh86+/+*^ and *aip* ^*wh239+/+*^ larvae had zero mutant transcripts and a mean of 271 and 180.75 WT transcripts, respectively. *aip* ^*wh86+/−*^ and *aip* ^*wh239+/−*^ larvae had nearly 100% WT transcripts (109 and 90.75 WT and four and 4.75 mutant, respectively). Again, we hypothesize that this is likely due to nonsense-mediated decay (NMD), a process that degrades aberrant transcripts, including those with early stop codons (Patro et al., 2024). *aip* ^*wh86−/−*^ and *aip* ^*wh239−/−*^ animals had mostly mutant transcripts, some WT transcripts, but very few transcripts overall (9.50 and 2 WT and 16.75 and 20.50 mutant, respectively), again suggesting NMD. Similar results were seen in a previous RNA-sequencing study using Aip mutant zebrafish (Karchner et al., 2026).

**FIGURE 5:**
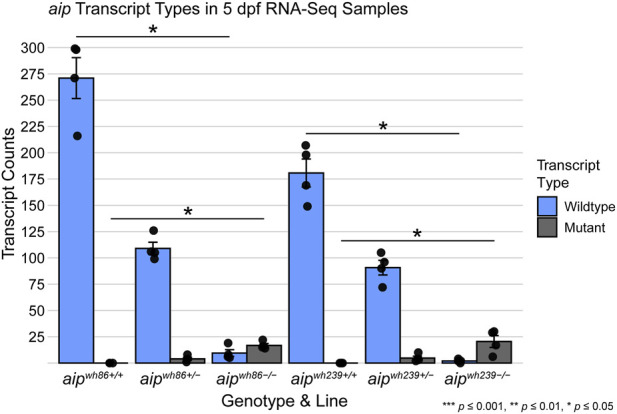
*aip* WT and mutant transcript counts in five dpf RNA-seq samples. Sample genotype and line are presented on the x-axis, and transcript counts from the integrated genome viewer (IGV) web app are on the y-axis. Transcript type is denoted by color. Individual sample counts correspond to the black circles overlayed on the bars. Significant differences between genotype are denoted by an asterisk, * (*p*-value ≤0.05, Kruskal–Wallis test followed by Dunn’s *post hoc* tests with Holm correction, error bars = standard error). Statistically significant differences in transcript abundance could only be found within line and between *aip* ^*+/+*^ *and aip* ^*−/−*^ larvae. However, it appears that *aip* ^*+/−*^ larvae have an intermediate number of WT transcripts and that mutant transcripts are largely degraded, likely through nonsense mediated decay. *aip* ^*−/−*^ larvae have some maternal WT transcripts, but very few *aip* transcripts overall.

Significant differences were found only when comparing WT and mutant transcript abundance between *aip* ^*+/+*^ *and aip* ^*−/−*^ larvae within a line. *aip* ^*+/−*^ larvae had intermediate values that were not distinguishable from the other genotypes. Additionally, significant differences could not be determined when comparing transcript abundance within genotypes and between lines. Figure 5 shows the mean number of each transcript type in each genotype and line. The black points are counts for each individual sample.

In the PCA (Section 3.3; Figure 4A), *aip* ^*wh86*−/−^ sample C3 diverged slightly from other *aip* ^*wh86*−/−^ samples and moved closer to *aip* ^+/+^ and *aip* ^+/−^ samples. This difference could also be observed in the sample-sample distance heatmap (Figure 4B) where sample C3 had lower distance to *aip* ^+/+^ and *aip* ^+/−^ samples compared to other *aip* ^*wh86*−/−^ fish. Hierarchical clustering still placed it with the three other *aip* ^*wh86*−/−^ samples and, more broadly, with all *aip* ^*−/−*^ samples. 23 genes from this sample also had Cook’s distances >1, indicating outlier counts for those genes. In Figure 5 C3 is represented by the point which is higher than the other three *aip* ^*wh86−/−*^ samples. This sample had a count of 19 maternal WT transcripts while the other three points averaged 6.3 transcripts. It should also be noted that replicates such as C3 are pools of five larvae, which reduces inter-replicate variation, but reduces individual-level resolution. All *aip* transcript counts can be found in Supplementary Dataset 3-Table 3.

### 3.5 Homozygous *aip* mutation alters pathways associated with cellular homeostasis

**FIGURE 6:**
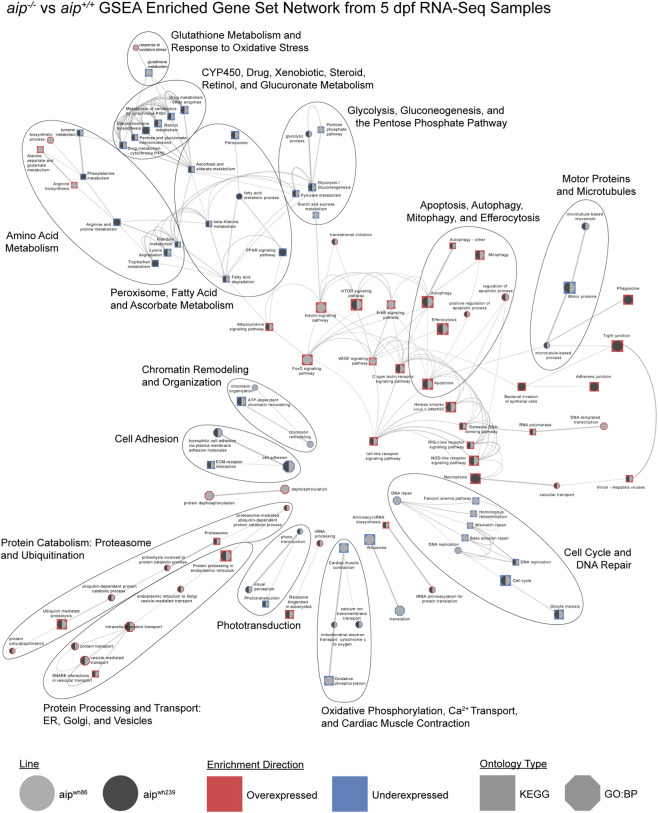
Network of KEGG and GO:BP terms enriched via GSEA (FDR ≤0.05) using *aip* ^*−/−*^ vs*.* aip^+/+^ gene expression changes from five dpf RNA-seq data. The network was generated by uploading GSEA results to the Enrichment Map app within Cytoscape. Line (*aip* ^*wh86*^ and/or *aip* ^*wh239*^) is denoted by the color (light and/or dark grey) of the foreground circle of each node. Enrichment direction (over or underexpression) is denoted by the color (red or blue) of the background shape of each node. Ontology type (KEGG or GO:BP) is denoted by the background shape (square or octagon) of each node. The size of the node and node connectors (edges) is determined by the number of genes in a gene set and the number of genes shared between nodes, respectively. 25% of genes in a smaller gene set must overlap with the larger gene set to be connected with an edge. Nodes with no connections were removed and are displayed in Figure 7. The ellipses were manually curated to highlight groups of three or more similar nodes. Individual gene set names can be viewed when the image is enlarged.

**FIGURE 7:**
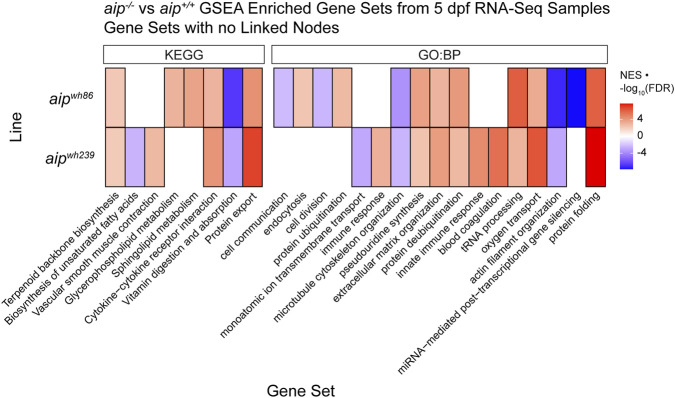
KEGG and GO:BP terms enriched via GSEA (FDR ≤0.05) using *aip* ^*−/−*^ vs*.* aip^+/+^ gene expression changes from five dpf RNA-seq data. While these gene sets were significantly enriched, they did not group with any other nodes in Figure 6 and were removed from the network. The terms are split by line and ontology type. The tile fill corresponds to normalized enrichment score (NES) · -log_10_(FDR). Red shaded tiles represent overexpression, while blue shaded tiles represent underexpression.

The GSEA enriched 129 unique pathways (FDR *q*-value ≤0.05): 77 KEGG, and 52 GO:BP terms. 78 of the terms were shared between the lines while 33 and 18 were unique to the *aip* ^*wh86*^ and *aip* ^*wh239*^ lines, respectively. All pathways that were positively or negatively enriched in one line were enriched in the same direction in the other. The range of normalized enrichment scores (NES) were |1.56–2.77| in either line. Within this network 35 GO:BP terms interconnected and bridged with the 69 KEGG terms, suggesting a high degree of concordance and consistency in the gene list. While 104 of the pathways connected to at least one other node in the network (Figure 6), 25 pathways had no connections and were displayed in a heatmap (Figure 7).

In Figure 6, groups of nodes with similar ontologies are circled and annotated to draw attention to their possible shared roles. Largely, these groups were enriched in the same direction and suggest coordinated shifts in pathways modulating nutrient metabolism, xenobiotic and lipid metabolism, protein trafficking, oxidative stress, the cell cycle, and programmed cell death. Often linked to these clusters were multiple KEGG signaling pathways including FoxO, mTOR, toll-like receptor, RIG-I-like receptor, insulin, ErbB, and VEGF which were all positively enriched. Pathways associated with apoptosis, autophagy, mitophagy, protein catabolism, and protein transport were positively enriched. Pathways associated with catabolic metabolism, DNA repair, chromatin remodeling, the cell cycle, microtubules, and xenobiotic, drug, steroid, and retinol metabolism were all negatively enriched. These changes suggest that in *aip* mutant zebrafish cellular stress response pathways are activated while proliferative pathways are suppressed, highlighting Aip’s critical roles beyond Ahr modulation. All significantly enriched GSEA gene sets can be found in Supplementary Dataset 5-Table 1.

### 3.6 MuSiC estimated cell-type-specific differences in transcriptional profile

**FIGURE 8:**
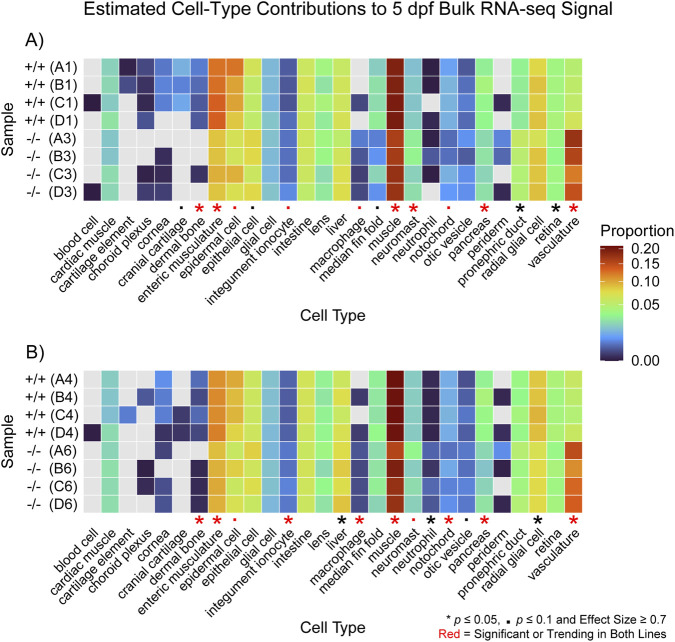
The contribution of different cell types to the five dpf, bulk RNA-seq data as estimated by MuSiC for the *aip* ^*wh86*^ **(A)** and *aip* ^*wh239*^ **(B)** lines. Cell types are presented on the x-axis, and genotype/sample ID is on the y-axis. The color bar representing proportion is log-scaled to better display contrast in low proportion cell types. Cell types with significant differences in proportion between *aip* ^*+/+*^ and *aip* ^*−/−*^ larvae are denoted with an asterisk, * (*p*-value ≤0.05, two-sided Wilcoxon rank sum test). Cell types trending towards significant differences were annotated with a period, (*p*-value ≤0.1, effect size greater than 0.7). Cell types that were significant or trending in both lines were denoted with a red asterisk or period. Differences in estimated cell-type contributions between genotypes may reflect transcriptomic changes within specific cell types associated with *aip* deficiency.

38 cell types were extracted from the single-cell atlas dataset after processing, however, 10 of these cell types (trunk, pectoral fin, fin, myoblast, pharyngeal arch, neuron, myotome, hematopoietic stem cell, central nervous system, and mesoderm) were not detected in any of the samples from the bulk RNA-seq data and were removed from Figure 8. These cell types may not have contributed enough cell-type-specific mRNA to the bulk mRNA pool to be detectable. This does not mean they were not present in the animal, but they may be in low abundance or have poor marker representation. PERMANOVA was used to test for global changes in cell-type composition. A significant *p*-value of 0.039 was reported for both the *aip* ^*wh86*^ and *aip* ^*wh239*^ lines with 77% and 76% of the variation being explained by genotype in each line, respectively.

Since both lines showed significant changes in global composition between genotypes, the Wilcoxon rank sum test was performed on both data sets. In the *aip* ^*wh86*^ line, 8 cell types exhibited statistically significant differences between genotypes (raw *p*-value ≤0.05), while 11 cell types were significant in the *aip* ^*wh239*^ line. Cell types trending towards significant differences were also annotated. Trending cell types had a *p*-value ≤0.1 and effect size ≥0.7, representing a large magnitude of change. Despite not being statistically significant, these cell types are still discussed, as many have sparse data for certain samples (grey cells), which could reduce the power of the Wilcoxon rank sum test.

In total, considering both lines, 19 cell types were either significant or trending, ten of which overlapped between the lines suggesting a degree of concordance. The remaining 9 cell types were unique to one line or the other. These changes in proportion should be interpreted as potential differences in transcriptional profile within specific cell types rather than a change in the actual composition of the animals. Cell types with significant shifts in transcriptional abundance as estimated by MuSiC could be grouped into several tissue categories. In one or both lines, these included structural tissues of mesodermal origin (dermal bone, muscle, notochord, and vasculature), neural tissues (glial cells, neuromast, and retina), immune cells (macrophage and neutrophil), and metabolic organs (liver, pancreas, and pronephric duct) (Schier and Talbot, 2005). MuSiC estimated cell-type proportions and statistics are available in Supplementary Dataset 5-Table 2.

### 3.7 Enriched transcription factor binding motifs control stress response, metabolic homeostasis, and cellular differentiation

Binding motif analysis was performed for four separate groups of *aip* ^*−/−*^ vs. *aip* ^*+/+*^ DEGs: higher abundance DEGS in the *aip* ^*wh86*^ line (↑*aip* ^*wh86*^), lower abundance in the *aip* ^*wh86*^ line (↓*aip* ^*wh86*^), higher abundance in the *aip* ^*wh239*^ line (↑*aip* ^*wh239*^), and lower abundance in the *aip* ^*wh239*^ line (↓*aip* ^*wh239*^). Overall, 33 unique TF binding motifs were enriched (adjusted *p*-value ≤0.05), representing twenty families and twelve classes.

**FIGURE 9:**
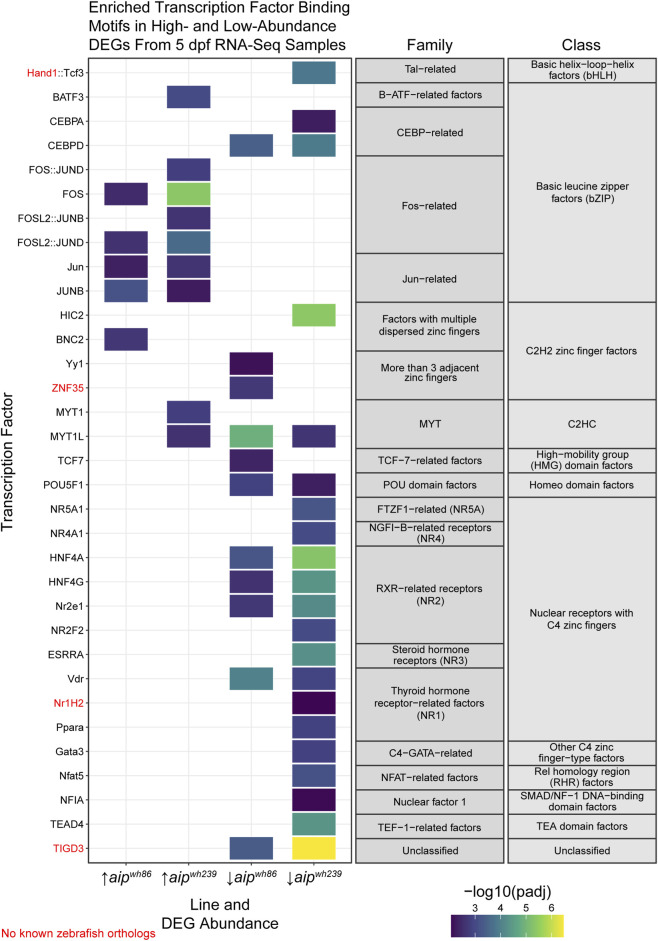
Enriched transcription factor (TF) binding motifs ±1,000 bp of the transcription start site in higher and lower abundance DEGs from the five dpf RNA-seq data (*p*adj ≤ 0.05, Fisher’s exact test with Bonferroni correction). Line and DEG abundance are on the x-axis, and the enriched TFs are on the y-axis. Family and class of each TF are annotated on the right. All TF information comes from the JASPAR 2026 vertebrate collection, and those enriched in our data were mice or human proteins. While most TFs had zebrafish orthologs, protein symbols in red have no known orthologs in ZFIN or Ensembl.

The ↓*aip* ^*wh239*^ group had the highest number of unique binding motifs (13). It shared seven motifs with the ↓*aip* ^*wh86*^ group which had only three unique motifs. Shared motifs were from the basic leucine zipper, C4 zinc finger nuclear receptor, homeodomain factor, or unclassified classes. They were members of the C/EBP-related, POU domain, NR2-RXR-related, or NR1-thyroid hormone-related receptor families (Figure 9). Most motifs enriched from genes with lower abundance were associated with nuclear receptor and developmental TFs including HNF4A/HNF4G, VDR, POU5F1, Nr2e1, and CEBPD which regulate metabolic homeostasis and cellular differentiation (Sladek, 2011; Hayhurst et al., 2001; Carlberg and Campbell, 2013; Nichols et al., 1998; Shi et al., 2004; Ramji and Foka, 2002).

The ↑*aip* ^*wh86*^ group had one unique binding motif. It shared four motifs with the ↑*aip* ^*wh239*^ group which had four unique motifs. Shared motifs were from the basic leucine zipper class and Fos and Jun-related families (Figure 9). Together these proteins form the activator protein 1 (AP-1) TF which is composed of dimers from the Jun-, Fos-, and ATF-related families. It regulates transcriptional response to growth factors, stress signals, infections, and in oncogenesis and tumor suppression (Eferl and Wagner, 2003; Hess et al., 2004). Zebrafish orthologs of these TFs, *junba*, *junbb*, *fosl2*, and *fosab*, were all DEGs in greater abundance in both lines while *batf3* was a DEG in greater abundance in the *aip* ^*wh239*^ line only. The only motif that was shared between higher and lower abundance DEGS was MYT1L, a MYT family and C2HC class TF.

The JASPAR 2026 vertebrate database did not contain any zebrafish binding motifs and the 33 unique motifs enriched in our samples were derived from either *H. sapiens* (humans) or *M. musculus* (mice). However, direct zebrafish orthologs were available for 29 of the motifs. Four had no direct zebrafish orthologs according to ZFIN (Bradford et al., 2022) and Ensembl: HAND1, NR2E1, TIGD3, and ZNF35. HAND1 was not listed individually but was included as a heterodimer with TCF3 which has multiple zebrafish orthologs. All TF binding motif stats tables can be found in Supplementary Dataset 5-Table 3.

### 3.8 Homozygous *aip* mutation alters transcript usage beyond differential expression

**FIGURE 10:**
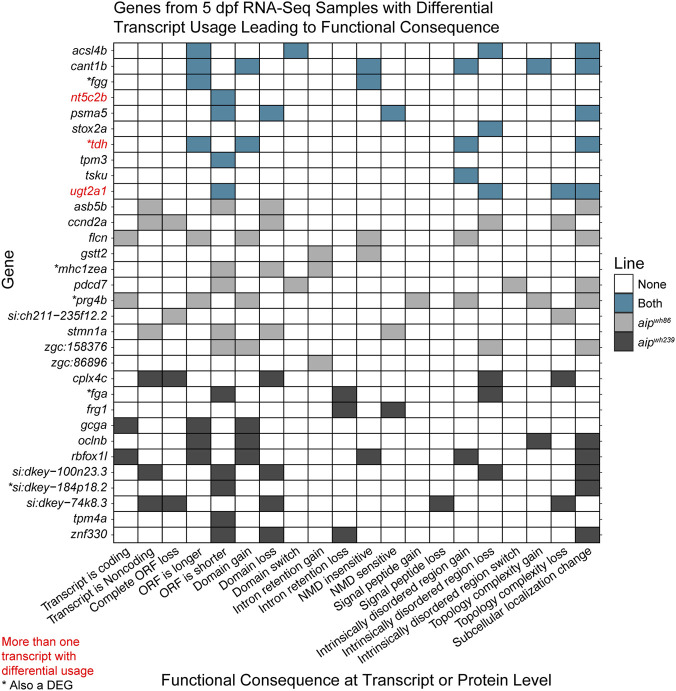
Genes with differential transcript usage (DTU) (Benjamini–Hochberg FDR-adjusted *q*-value ≤0.05) from the five dpf RNA-seq data. The predicted functional consequence of DTUs with isoform fraction (dIF) ≥ |0.1| is on the x-axis, and the gene expressing each DTU is on the y-axis. These functional consequences correspond to the structure of the transcript with increased abundance in *aip* ^*−/−*^ larvae vs. the more abundant transcript in *aip* ^*+/+*^ larvae. Genes with more than one transcript in differential usage are in red. Genes that are also DEGs are denoted by an asterisk, *. 32 genes had DTUs that were predicted to result in functional consequences at the transcript or protein level, with 10 shared between the lines. Each line had 11 unique genes with DTU. Only six of the DTU genes were also DEGs.

36 and 35 genes with significant differences in transcript usage between *aip* ^*+/+*^ and *aip* ^*−/−*^ larvae were identified (*q* ≤ 0.05) from the *aip* ^*wh86*^ and *aip* ^*wh239*^ lines, respectively. Only 17 were shared between lines. In both lines, 32 genes were estimated to have differential transcript usage with functional consequences at the protein level in one or both lines (Figure 10). 10 of these genes were shared between lines. These changes represent the alterations in transcripts or translated protein with greater expression in *aip* ^*−/−*^ larvae vs. the transcripts with greater expression in *aip* ^*+/+*^ larvae. Out of the 11 DTU genes in the *aip* ^*wh86*^ line only two were also DEGs. Out of the 11 DTU genes in the *aip* ^*wh239*^ line only two were also DEGs. Out of the 10 DTU genes shared between lines again just two were also DEGs. The DTU consequences and stats tables are in Supplementary Dataset 5-Table 4.

DTU genes that were found in one or both lines were placed into g:Profiler (Kolberg et al., 2023) for pathway enrichment. When adding shared DTU genes only the KEGG pathway “pyrimidine metabolism” was enriched due to genes calcium activated nucleotidase 1 b (*cannot1b*) and 5′-nucleotidase, cytosolic IIb (*nt5c2b*). The WikiPathways (Agrawal et al., 2024) term “effect of L-carnitine on metabolism” was also enriched due to acyl-CoA synthetase long chain family member 4 b (*acsl4b*). When using the shared DTU genes plus DTU genes only present in the *aip* ^*wh86*^ line no pathways were enriched. When adding shared DTU genes plus DTU genes only present in the *aip* ^*wh239*^ line GO:BP terms “protein activation cascade”, “blood coagulation, fibrin clot formation”, “platelet aggregation”, and GO:CC term “fibrinogen complex” were enriched due to fibrinogen chains alpha and gamma (*fga*, *fgg*). Additionally, the WP pathway “effect of L carnitine on metabolism” was still enriched due to *acsl4b*.

## 4 Discussion

Morphological and behavioral assessments were performed on five dpf *aip* ^*wh239−/−*^, *aip* ^*wh239+/−*^, and *aip* ^*wh239+/+*^ larvae following exposure to four Ahr ligands. We observed that *aip* ^*wh239−/−*^ larvae had some degree of differential susceptibility vs. the other genotypes during each of the exposures. Susceptibility was ligand-dependent, with *aip* ^*wh239−/−*^ larvae being less susceptible to PCB126, 5-Nan, and BkF, but more sensitive to leflunomide exposure. Further, Ahr ligands increased the incidence of similar morphological endpoints, but *aip* mutation altered susceptibility to these malformations in some chemicals but not others.

We performed mRNA sequencing of pooled five dpf larvae from each genotype and line. Because RNA-seq samples represent pooled larvae, transcriptomic results do not resolve inter-individual transcriptional variability within genotype groups. As such, results should be interpreted as genotype-associated differences across pooled biological replicates rather than individual-level responses. While WT maternal transcripts were present in *aip* ^*−/−*^ mutants, there were large transcriptomic changes, including differential expression and alternative splicing, due to *aip* deficiency. GSEA revealed that pathways related to cellular and metabolic homeostasis were disturbed in *aip* ^*−/−*^ larvae, and enrichment of DEG binding motifs estimated changes in binding activity by transcription factors such as nuclear receptors and AP-1, which regulate metabolic homeostasis, cellular differentiation, and stress, that may be tied to these transcriptomic phenotypes. Because homozygous *aip* mutation is lethal by 10 dpf some genotype-dependent differences observed at five dpf may be the downstream result of impaired development or cellular stress rather than a direct mechanistic consequence of Aip mutation. Due to this, RNA-seq results should be interpreted as changes associated with Aip deficiency, not direct regulatory changes due to reduced Aip activity.

Finally, we used MuSiC to estimate alterations in transcriptional profile within specific cell types. This analysis increased the resolution of the bulk RNA-seq data by estimating the transcriptomic contributions of 28 different cell types. MuSiC-based estimates in proportion were available only for cell types with sufficient representation in the single-cell atlas and the bulk RNA-seq data. 38 cell types were retained from the five dpf reference after filtering, and 10 of these cell types had no estimated contribution for the bulk samples. These populations may be in low abundance or contribute little cell-type-specific mRNA. Due to this, estimates are available for retained cell types only, rather than all possible cell types comprising a larval zebrafish. Changes in proportion for retained cell types should be interpreted as potential cell-type-specific differences in transcriptional profile rather changes in the composition of larvae.

### 4.1 *aip* status strongly influences phenotype while line-to-line differences are less clear

We demonstrated that *aip* deficiency altered susceptibility to Ahr ligands and unexposed larval behavior (Figures 1–3). Using mRNA-sequencing we found many DEGs between *aip* ^*−/−*^ and *aip* ^*+/+*^ larvae in both lines, as well as alternative splicing products which were not DEGs (Figure 10). In contrast, *aip* ^*+/−*^ larvae appeared to be nearly identical to *aip* ^*+/+*^ larvae transcriptomically, with less than six DEGs in either line (Table 1). This is despite the fact that five dpf *aip* ^*+/−*^ larvae have around half as many WT *aip* transcripts as *aip* ^*+/+*^ larvae, suggesting that lower expression of WT *aip* transcripts is sufficient to maintain normal cellular function (Figure 5). Additionally, *aip* ^*+/−*^ larvae were not significantly different from *aip* ^*+/+*^ larvae in any of the morphological screenings performed following Ahr ligand exposure. There is strong evidence that heterozygous *Aip* loss in mice leads to excess growth hormone (Schernthaner-Reiter et al., 2020) and pituitary tumors (Lecoq et al., 2016; Shen et al., 2024) with one study demonstrating full penetrance of the phenotype by the age of 15 months (Raitila et al., 2010). While normal phenotypes were observed in *aip* ^*+/−*^ larval zebrafish, future studies in *aip* ^*+/−*^ adults may find more subtle effects due to heterozygous *aip* mutation.

Five dpf *aip* ^*−/−*^ larvae had low levels of both WT and mutant *aip* transcripts, with WT transcripts present at 3.5% and 1.1% of the level observed in *aip* ^*+/+*^ larvae for the *aip* ^*wh86*^ and *aip* ^*wh239*^ lines, respectively. This low abundance of WT *aip* transcripts appears insufficient for normal development, with reduced survival of *aip* ^*−/−*^ larvae after five dpf. However, this data excludes earlier developmental timepoints, where maternal transcript carryover could partially rescue the initial effects of homozygous aip deficiency. Consideration of earlier timepoints is important when interpreting Ahr ligand exposures, which began at six hpf. Maternal transcript carryover could also delay the onset of developmental phenotypes and influence transcriptomic states at five dpf. Karchner et al. (2026) examined the presence of maternal WT transcripts at three dpf in addition to five dpf. For *aip* ^*−/−*^ larvae at both time points, WT transcripts were present at ≈3% of the level observed in *aip* ^*+/+*^ larvae (Karchner et al., 2026). While low at three and five dpf, no data concerning maternal transcript counts is available at earlier developmental time points, and results may be interpreted as phenotypes associated with severe Aip deficiency, rather than complete loss.

Differences between the lines were more difficult to interpret, as baseline transcriptomic changes were present between *aip* ^*wh86+/+*^ and *aip* ^*wh239+/+*^ larvae (105 DEGSs). Functional profiling of these DEGs performed using g:Profiler did not identify enriched pathways, supporting the hypothesis that this divergence is due to the lines originating from different founders or from maintenance as separate stocks rather than a coordinated change in gene expression. Therefore, in this study we used the convergence between the two lines to support shared conclusions rather than attempting to explain differences in phenotype between the mutant lines. This approach is further supported by the fact that the *aip* mutants had very few mutant transcripts overall (mean ≈19) and that death occurred within the same developmental time point in both lines (Karchner et al., 2026). However, functional profiling of *aip* ^*wh239−/−*^ vs. *aip* ^*wh86−/−*^ DEGs using g:Profiler did reveal some enriched pathways, mostly involving steroid biosynthesis, heme binding, retinol metabolism, and PPAR signaling. Additionally, we cannot exclude the possibility that mutant gene products retain partial activity. This is especially true for *aip* ^*wh239−/−*^ animals, as a single TPR domain is encoded in the translated protein. If any mutant protein is translated, it is possible that it could interact with other proteins through this domain in very specific and limited circumstances. Again, because of this, results may be associated with severe Aip deficiency, rather than complete loss of Aip activity.

### 4.2 Homozygous *aip* mutation may protect against ahr-dependent toxicity while increasing susceptibility to Ahr-independent exposures

PCB126 is a dioxin-like compound with Ahr2-dependent toxicity in zebrafish (Jonsson et al., 2007). Previously, killifish with adaptations to chronic PCB exposure (Reid et al., 2016) and Aip mutant zebrafish (Karchner et al., 2026) exhibited resistance to PCB126 as well as TCDD. In this study, we expanded knowledge of Aip-mediated chemical sensitivity by exposing *aip* mutant zebrafish to three additional Ahr ligands: 5-nitroacenaphthene (5-Nan), benzo(k) fluoranthene (BkF), and leflunomide. To our knowledge, these Ahr ligands have never been tested in an *aip*-null model.

Each of these Ahr ligands have properties not observed with dioxin-like compounds previously tested. First, where many dioxin-like compounds bind to Ahr2 in zebrafish (Andreasen E. A. et al., 2002), the substituted PAH 5-Nan was shown to be a presumptive Ahr1a-specific ligand (Morshead et al., 2025). Second, a previous study found that in a screen of 123 PAHs, four induced a duplicate fin fold perpendicular to the caudal fin that was coined “X-fin”. BkF was the most potent of the four PAHs. The X-fin phenotype only occurred in larvae exposed before 36 hpf and was Ahr2-dependent (Garland et al., 2020). Third, the anti-inflammatory prodrug leflunomide was also tested. While leflunomide is an Ahr2 agonist, its rapid *in vivo* metabolism to therapeutic agent A771726 (teriflunomide) is largely Ahr2-independent, with CYP enzymes only playing a small role (Rozman, 2002). Teriflunomide inhibits DHODH, a mitochondrial enzyme which catalyzes the rate-limiting step of *de novo* pyrimidine synthesis, critical in dividing cells (Sykes, 2018). Teriflunomide does not activate Ahr2. While in the present study *aip* deficiency sensitized zebrafish larvae to leflunomide, Ahr2 was shown to be required for leflunomide-induced inhibition of caudal fin regeneration in zebrafish (O'Donnell et al., 2010). Leflunomide also binds Ahr1a and Ahr1b, but their spatial expression is less broad than Ahr2, with Cyp1a induction in leflunomide-exposed *ahr2* ^*hu3335*^ larvae largely confined to the liver and vasculature. *ahr1a* knockdown blocked Cyp1a expression in the liver and *ahr1b* knockdown blocked expression in the vasculature (Goodale et al., 2012).

While both PCB126 and 5-Nan exposure led to cranial and edema malformations in five dpf zebrafish larvae, *aip* mutation was only protective against these phenotypes during PCB126 exposure (Figure 1). However, the brain endpoint, which was induced during 5-Nan exposure, had significantly lower incidence in 5-Nan-exposed *aip* ^*wh239−/−*^ larvae. BkF exposure led to significant induction of cranium and edema endpoints, but to a lesser degree than the PCB126 and 5-Nan exposures. As with 5-Nan exposure, *aip* mutation was not protective against these phenotypes following BkF exposure. The endpoint primarily driving BkF toxicity in zebrafish larvae was the X-fin caudal fin duplication and *aip* ^*wh239−/−*^ larvae had significantly lower incidence of this phenotype. Together, these results suggest Ahr isoform-, chemical-, and tissue-specific modulation of Ahr by Aip.

For both the 5-Nan and BkF exposures, *aip* ^*wh239−/−*^ larvae had increased incidence of axis malformations vs. *aip* ^*wh239+/+*^ or *aip* ^*wh239+/−*^ larvae, respectively. This apparent increase in sensitivity to axis malformations may be explained by reduced or delayed hatching that is sometimes observed in *aip* ^*wh239−/−*^ zebrafish. Delayed hatching was not quantified in this study, as hatching occurs around three dpf and morphology was not observed until five dpf. Delayed hatching can lead to bends along the axis. There was also increased incidence of brain malformations in *aip* ^*wh239−/−*^ vs. *aip* ^*wh239+/−*^ larvae during the BkF exposure. When comparing the DMSO and 40 µM BkF-exposed *aip* ^*wh239−/−*^ larvae there was no significant difference in incidence of axis or brain endpoints, suggesting these are background malformations. Work utilizing brightfield microscopy to better characterize *aip*-dependent morphological and tissue malformations absent chemical exposure has been performed and is in preparation (Stinson et al., 2026, in preparation) at the time of the current study’s publication.

Behavioral results from exposed *aip* ^*wh239−/−*^ larvae agreed with or differed from the paired morphologic data depending on the Ahr ligand tested. PCB126-exposed *aip* ^*wh239−/−*^ larvae had no significant behavioral effect, while both *aip* ^*wh239+/+*^and *aip* ^*wh239+/−*^ larvae were hypoactive during the light phase of the assay. However, the genotype • exposure interactions were not significant, suggesting that, PCB126 exposure did not produce significantly greater hypoactivity in *aip* ^*wh239+/+*^ and *aip* ^*wh239+/−*^ larvae vs. *aip* ^*wh239−/−*^ mutants. Following 5-Nan exposure, all genotypes were hypoactive in both the light and dark phases of the LPR assay and exposure induced greater hypoactivity in *aip* ^*wh239+/−*^ larvae vs. *aip* ^*wh239−/−*^ larvae. However, 5-Nan-induced behavioral alterations also differed between *aip* ^*wh239+/+*^ and *aip* ^*wh239+/−*^ larvae, while *aip* ^*wh239+/+*^ and *aip* ^*wh239−/−*^ behavioral alterations were not statistically different. This makes the role of *aip* deficiency in 5-Nan-induced behavioral alterations more difficult to interpret, but the likely minimal protection conferred by *aip* mutation is consistent with the morphological data. However, one may hypothesize that reduction in 5-Nan-induced brain malformations could affect behavioral readouts. Similarly, all BkF-exposed genotypes were hypoactive in the light and dark phases of the LRP assay and during the LSR assay. Yet, none of the genotype • exposure interactions were statistically significant. This suggests that while *aip* mutation prevents BkF-induced caudal fin duplications, it does not protect against BkF-induced behavior effects. The complex behavioral effects seen following exposure to different Ahr ligands further highlight the tissue-specific roles of Aip in Ahr modulation.

The effect of leflunomide exposure on *aip* ^*wh239−/−*^ larvae contrasts with the other three Ahr ligands tested. Rather than offering resistance, *aip* mutation led to more severe morphological and behavior effects during leflunomide exposure (Figures 1, 3). Leflunomide is distinct from the other AHR ligands, as it can act through both Ahr induction and Ahr-independent conversion to teriflunomide, the active metabolite responsible for inhibition of DHODH. Our results contrasts with O’Donnell et al., 2010 where Ahr knockdown rescued leflunomide-induced inhibition of caudal fin regeneration. In O’Donnell et al., 2010, it was concluded that DHODH inhibition was not responsible for fin regeneration inhibition, as direct teriflunomide exposure did not block fin regeneration in Ahr competent larvae. We postulate that leflunomide-induced toxicity in *aip*-null zebrafish was largely due to Ahr-independent formation of teriflunomide and downstream DHODH inhibition (Rozman, 2002), while toxicity of the three other ligands are known to be mediated through AHR signaling. Additionally, we observed dysregulation of mitochondrial and oxidative stress-related pathways in *aip* ^*wh239−/−*^ larvae, consistent with altered cellular homeostasis. We hypothesize, that due to baseline cellular stress, *aip* ^*wh239−/−*^ larvae may have a diminished ability to respond to leflunomide or its metabolites, with reduced Ahr signaling insufficient to prevent toxicity. Specifically, we hypothesize that teriflunomide exacerbates cellular stress through further inhibition of the mitochondrial function or *de novo* pyrimidine synthesis, a process critical in dividing cells (Sykes, 2018). Further, Ahr knockout mice were shown to have higher blood teriflunomide levels and worsened toxicity compared to WT mice, suggesting that Ahr-mediated induction of xenobiotic-metabolizing enzymes is important in clearance of the drug (Redaelli et al., 2015). Together, these observations suggest that reduced Aip activity may be insufficient to decrease leflunomide toxicity, and may even be detrimental due to increased cellular stress and reduced teriflunomide clearance. To test these hypotheses, future experiments should determine whether direct teriflunomide exposure induces morphological and behavioral effects such as those seen following leflunomide exposure. Additionally, it would be informative to repeat the caudal fin regeneration experiment performed in O’Donnell et al. (2010) using *aip* mutant zebrafish.

### 4.3 Coordinated shifts in transcription promote stress and immune response while suppressing metabolic pathways

DEG analysis revealed widespread transcriptomic changes in *aip* ^*−/−*^ larvae from both the *aip* ^*wh86*^ and *aip* ^*wh239*^ lines at five dpf and regulatory and cell-type-specific consequences associated with *aip* mutation were inferred using GSEA, TF binding motif analysis, and bulk-RNA-seq deconvolution. Genes in pathways associated with the cell cycle, DNA replication and repair, nutrient and drug metabolism, and the mitochondria and electron transport chain (ETC.) were primarily in lower abundance. In contrast, genes associated with cellular stress, apoptosis, innate immune response, and protein ubiquitination and transport were primarily in greater abundance (Figures 6, 7). Together, these findings raise the possibility that coordinated shifts in transcription promote cellular stress responses and repress proliferation and maintenance, disrupting cellular homeostasis.

Multiple gene sets associated with glycolysis, the pentose phosphate pathway, and amino acid and fatty acid metabolism were negatively enriched, suggesting that cellular metabolism is broadly reduced. ETC-related terms “mitochondrial electron transport, cytochrome c to oxygen” and “oxidative phosphorylation” were negatively enriched, suggesting that metabolic dysfunction extends to mitochondrial ATP production. The “PPAR signaling pathway” was also negatively enriched. PPAR signaling regulates genes involved in lipid metabolism, adipogenesis, and energy homeostasis (Tyagi et al., 2011). Notably, some gene sets involved in protein metabolism and biosynthesis, “alanine, aspartate, and glutamate metabolism”, “arginine biosynthesis”, and “biosynthetic process”, were positively enriched, suggesting that certain metabolic processes may be maintained despite *aip* mutation. Additionally, many pathways associated with drug and xenobiotic metabolism were negatively enriched as were “steroid hormone biosynthesis” and “retinol metabolism” gene sets. AME was used to enrich TF binding motifs among genes of lower abundance and enriched HNF4A and HNF4G, TFs that regulate lipid, glucose, and xenobiotic metabolism (Bolotin et al., 2010; Beinsteiner et al., 2023; Hwang-Verslues and Sladek, 2010). MuSiC-based bulk-RNA-seq deconvolution estimated decreased transcriptomic contributions from the pancreas between *aip* ^*−/−*^ and *aip* ^*+/+*^ larvae in both lines (Figure 8). *hnf4a* is expressed in pancreatic β-cells of the developing zebrafish (O'Hare et al., 2016). POU5F1 and NR2E1 motifs were also enriched from lower abundance genes, suggesting potential alterations in pathways that control differentiation and proliferation (Onichtchouk, 2012; Onichtchouk et al., 2010; Kitambi and Hauptmann, 2007), however their role here remains unclear.

Cellular stress response pathways involving apoptosis, proteolysis, and oxidative stress were positively enriched. In both lines, “apoptosis”, “regulation of apoptotic process”, and “positive regulation of apoptotic response” gene sets were enriched, alongside “autophagy”, “efferocytosis”, and “mitophagy”. Additionally, gene sets associated with protein ubiquitination, proteolysis, and protein transport in the endoplasmic reticulum and Golgi apparatus were positively enriched in both lines. “Response to oxidative stress” was positively enriched in the *aip* ^*wh86*^ line only. These coenriched pathways may be the result of increased turnover of damaged or misfolded proteins, recycling of organelles, such as the mitochondria, and programmed cell death and efferocytosis, possibly in response to elevated cellular stress. Interestingly, Aip is known to bind with certain mitochondrial proteins, including survivin, which is tightly regulated in several cellular compartments. In the cytosol it prevents maturation of proapoptotic caspases, inhibiting apoptotic events. Survivin also regulates cell division by helping to segregate chromosome during mitosis (Martini et al., 2016; Albadari and Li, 2023). In the mitochondria it prevents intrinsic apoptosis and cytochrome C release (Malcles et al., 2007; Dohi et al., 2004). Reduction of survivin levels in the cell and mitochondria could potentially lower apoptotic threshold and inhibit mitosis. Survivin-null mice do not survive beyond E4.5, while *Aip*-null lines see 100% lethality at E13.5–14 (Uren et al., 2000; Kang et al., 2011). AIP also interacts with the rearranged during transfection (RET) proto-oncogene, which interferes with AIP-survivin interaction, acting as a potential regulatory pathway in cell survival (Vargiolu et al., 2009; Garcia-Rendueles et al., 2021). *RET* null mice have developmental abnormalities and die shortly after birth (Schuchardt et al., 1994).

Among genes with greater abundance in *aip* ^*−/−*^ larvae, enriched TF binding motifs were primarily from the Jun-, Fos-, and ATF-related families, which dimerize to form the AP-1 TF (Figure 9). Zebrafish orthologs for these genes were also in greater abundance, supporting these results. Additionally, innate immune signaling-related gene sets, “RIG-I-like receptor signaling pathway”, “NOD-like receptor signaling pathway”, “C-type lectin receptor signaling pathway”, “toll-like receptor signaling pathway”, “immune response”, and “innate immune response”, were positively enriched. These innate immune pathways, known as pattern-recognition receptors (PRRs), activate downstream signaling cascades that drive innate immune response (Li and Wu, 2021; Yoneyama et al., 2024). Specifically, the toll-like receptor is known to activate AP-1 through MAPK signaling (Takeda and Akira, 2005; Wicherska-Pawlowska et al., 2021). Further, mitochondrial damage and reactive oxygen species have been shown to activate the innate immune system through PRRs (Chandel, 2014; West and Shadel, 2017). In *aip* ^*−/−*^ larvae, MuSiC-based bulk-RNA-seq deconvolution estimated increased transcriptomic contributions from macrophages in both lines and neutrophils in the *aip* ^*wh239*^ line, suggesting enhanced innate immune activation in *aip* ^*−/−*^ larvae. While these analyses identified processes and cell types overrepresented among the DEGs, results should be interpreted as inferential to generate mechanistic hypotheses and prioritize pathways requiring further validation.

## 5 Conclusion

*aip* mutation in larval zebrafish reduced sensitivity to toxicants whose activity depends on Ahr signaling. In contrast, *aip* ^wh239−/−^ larvae were more sensitive to leflunomide, an Ahr ligand that does not require Ahr activation to drive toxicity. Effects were also observed absent chemical exposure, including hyperactivity in the light phase of the LPR assay and early mortality at 7–10 dpf. Large transcriptomic shifts were observed in *aip* ^*−/−*^ larvae, including alternative splicing. Gene expression changes were used to perform GSEA, indicating positively enriched gene sets involved in cellular stress, apoptosis, innate immune response, and proteolysis, and negatively enriched gene sets involved in replication and metabolism. These observations were supported by enrichment of binding motifs governing many of these processes and estimates of transcriptomic contributions from important cell types. While these observations are inferential, and do not prove direct mechanistic claims, they suggest a context-dependent role of Aip in Ahr modulation and other cellular functions which may be demonstrated following future validation. As Aip function is critical for survival in zebrafish and other organisms, further studies could benefit from models that preserve Aip function but introduce targeted mutations. This approach would allow investigation of Aip’s role in later developmental stages, simplify methodology, and eliminate the potential impact of maternal WT transcripts.

## Data Availability

The datasets presented in this study can be found in online repositories. The names of the repository/repositories and accession number(s) can be found in the article/Supplementary Material.
