# Supplementary material for: Aip mutation leads to significant alterations in gene expression, altered sensitivity to Ahr ligands, and early lethality in zebrafish
Source: Front Toxicol. 2026 Jul 6;8:1875384. doi: 10.3389/ftox.2026.1875384 (PMC13381021; doi:10.3389/ftox.2026.1875384)
Supplement: Supplementary file 4 [file DataSheet1.docx]

Table or Figure Page Number

[Table 1 1](#_Toc228285906)

[Table 2 2](#_Toc228285907)

[Table 3 4](#_Toc228285908)

[Table 4 4](#_Toc228285909)

Table 1

Sequences of guide targets used to generate each line, the screening primers used for initial mutation detection, and the TaqMan primers and probe used for genotyping offspring.

|  |  | *aip^wh86^* | *aip^wh239^* |
| --- | --- | --- | --- |
| Target/Primer/Probe (5'-3') | Guide Target | GGTTACCACTATGAAAGAGG | AGCGTCCAGGTGATGAGCAC |
|  | Screening Fwd | GTGAAGTTTCATTACCGCACTAGC | CAATAACACCGTCGCTTTTGTTGC |
|  | Screening Rev | AAAGAATAAGAGTCCAGACAGACC | TAGTCATGTAGTGTGAAAACATCC |
|  | TaqMan Fwd | TGTGTGGGAGCAAGTGGTTAC | GGTTGAACATTAAACATCTCCCCTGTT |
|  | TaqMan Rev | ACAGCTAGAAATGTCACTGACCTTAAC | GGAGGAGAGGAGTGATCATGAGAT |
|  | TaqMan WT Probe | TTCCCCCTCTTTCATAGTG | ATGAGCACTGGATCAA |
|  | TaqMan Mutant Probe | ATTTCCCCCTTTCATAGTG | CAGGTGATGAGGATCAA |

Table 2

Chemical names, CAS numbers, purity, vendors, and stock concentrations for chemicals used. Additionally, the chemical, concentrations, DMSO normalization, and the samples sizes for all exposures (range finding, EC_50_ morphology, and EC_50_ behavior experiments).

| **Chemical Information** | | | | |  |  |  |
| --- | --- | --- | --- | --- | --- | --- | --- |
| **Chemical Name** | **CAS** | **Purity (%)** | **Vendor** | **Stock Concentration (mM)** |  |  |  |
| PCB126 | 57465-28-8 | 99.5 | AccuStandard | 0.382 |  |  |  |
| 5-nitroacenaphthene | 602-87-9 | 84.1 | AccuStandard | 10.14 |  |  |  |
| benzo(k)fluoranthene | 207-08-9 | 99.9 | AccuStandard | 10 |  |  |  |
| leflunomide | 75706-12-6 | ≥98 | Sigma-Aldrich | 10 |  |  |  |
|  |  |  |  |  |  |  | |
| **Range Finding** | | | | | | | |
| **Chemical Name** | **DMSO Normalization (%)** | **Conc 1 (µM)** | **Conc 2 (µM)** | **Conc 3 (µM)** | **Conc 4 (µM)** | **Conc 5 (µM)** | |
| PCB126 | 0.0785 | 0.1 | 0.15 | 0.2 | 0.25 | 0.3 | |
| 5-nitroacenaphthene | 0.1972 | 4 | 8 | 12 | 16 | 20 | |
| benzo(k)fluoranthene | 0.6000 | 20 | 30 | 40 | 50 | 60 | |
| leflunomide | 0.0100 | 0.6 | 0.7 | 0.8 | 0.9 | 1 | |
| **Chemical Name** | **n_DMSO** | **n_Conc 1** | **n_Conc 2** | **n_Conc 3** | **n_Conc 4** | **n_Conc 5** | |
| PCB126 | 64 | 64 | 64 | 64 | 64 | 57 | |
| 5-nitroacenaphthene | 48 | 48 | 48 | 48 | 48 | 48 | |
| benzo(k)fluoranthene | 48 | 48 | 48 | 48 | 48 | 48 | |
| leflunomide | 48 | 48 | 48 | 48 | 48 | 47 | |
|  |  |  |  |  |  |  | |
| **EC50 Exposure -Morphology** | | | | | | | |
| **Chemical Name** | **CAS** | **Purity (%)** | **Vendor** | **Stock Concentration (mM)** | **Exposure Concentration (µM)** | **DMSO Normalization (%)** | |
| PCB126 | 57465-28-8 | 99.5 | AccuStandard | 0.382 | 0.2 | 0.0524 | |
| 5-nitroacenaphthene | 602-87-9 | 84.1 | AccuStandard | 10.14 | 11 | 0.1085 | |
| benzo(k)fluoranthene | 207-08-9 | 99.9 | AccuStandard | 10 | 40 | 0.4000 | |
| leflunomide | 75706-12-6 | ≥98 | Sigma-Aldrich | 10 | 0.75 | 0.0075 | |
| **Chemical Name** | **n_+/+ DMSO** | **n_+/+ Exposed** | **n_+/- DMSO** | **n_+/- Exposed** | **n_-/- DMSO** | **n_-/- Exposed** | |
| PCB126 | 58 | 74 | 135 | 136 | 74 | 65 | |
| 5-nitroacenaphthene | 68 | 63 | 135 | 123 | 70 | 83 | |
| benzo(k)fluoranthene | 63 | 62 | 144 | 128 | 72 | 52 | |
| leflunomide | 64 | 61 | 162 | 134 | 56 | 50 | |

|  |  |  |  |  |  |  |
| --- | --- | --- | --- | --- | --- | --- |
| **EC50 Exposure - Behavior** | | | | | | |
| **Chemical Name** | **CAS** | **Purity (%)** | **Vendor** | **Stock Concentration (mM)** | **Exposure Concentration (µM)** | **DMSO Normalization (%)** |
| PCB126 | 57465-28-8 | 99.5 | AccuStandard | 0.382 | 0.2 | 0.0524 |
| 5-nitroacenaphthene | 602-87-9 | 84.1 | AccuStandard | 10.14 | 11 | 0.1085 |
| benzo(k)fluoranthene | 207-08-9 | 99.9 | AccuStandard | 10 | 40 | 0.4000 |
| leflunomide | 75706-12-6 | ≥98 | Sigma-Aldrich | 10 | 0.75 | 0.0075 |
| **Chemical Name** | **n_+/+ DMSO** | **n_+/+ Exposed** | **n_+/- DMSO** | **n_+/- Exposed** | **n_-/- DMSO** | **n_-/- Exposed** |
| PCB126 | 57 | 43 | 135 | 86 | 73 | 53 |
| 5-nitroacenaphthene | 68 | 30 | 135 | 62 | 70 | 48 |
| benzo(k)fluoranthene | 62 | 32 | 144 | 62 | 71 | 38 |
| leflunomide | 64 | 36 | 162 | 75 | 50 | 16 |
| No Exposure | 251 | NA | 576 | NA | 264 | NA |

Table 3

Description of each morphological endpoint assessed at 120 hpf following chemical exposure.

| Endpoint | Definition (at 5 dpf) |
| --- | --- |
| Cranium | Malformed, missing or smaller than normal the eye, snout, or jaw |
| Axis | Curved or bent axis in either direction |
| Edema | Heart or yolk sac malformation, pericardial or yolk sac edema |
| Muscle | Lack of circulation, malformed/disorganized/missing somites, or improper swim bladder formation |
| Lower Trunk | Malformation of the lower trunk, including caudal fin region |
| Brain | Brain malformations or necrosis |
| Skin | Abnormal pigmentation |
| Notocord | Notochord malformation |
| Touch Response | Not responsive to touch |
| X-Fin | Caudal fin duplication |

Table 4

Post-hoc pairwise comparisons performed for the four EC_50_ exposures to evaluate genotype and concentration effects. The null hypothesis tested by each comparison is provided as well.

| Condition 1 | Genotype 1 | Condition 2 | Genotype 2 | Null Hypothesis Tested |
| --- | --- | --- | --- | --- |
| DMSO | *aip^wh239+/+^* | DMSO | *aip^wh239+/-^* | Controls from each genotype behave the same |
| DMSO | *aip^wh239+/+^* | DMSO | *aip^wh239-/-^* |  |
| DMSO | *aip^wh239+/-^* | DMSO | *aip^wh239-/-^* |  |
| DMSO | *aip^wh239+/+^* | Exposed | *aip^wh239+/+^* | Chemical exposure does not have an effect |
| DMSO | *aip^wh239+/-^* | Exposed | *aip^wh239+/-^* |  |
| DMSO | *aip^wh239-/-^* | Exposed | *aip^wh239-/-^* |  |
| Exposed | *aip^wh239+/+^* | Exposed | *aip^wh239+/-^* | Exposed larvae from each genotype behave the same |
| Exposed | *aip^wh239+/+^* | Exposed | *aip^wh239-/-^* |  |
| Exposed | *aip^wh239+/-^* | Exposed | *aip^wh239-/-^* |  |
